# An evaluation of Minor Groove Binders as anti-*Trypanosoma brucei brucei* therapeutics

**DOI:** 10.1016/j.ejmech.2016.03.064

**Published:** 2016-06-30

**Authors:** Fraser J. Scott, Abedawn I. Khalaf, Federica Giordani, Pui Ee Wong, Sandra Duffy, Michael Barrett, Vicky M. Avery, Colin J. Suckling

**Affiliations:** a WestCHEM Department of Pure and Applied Chemistry, University of Strathclyde, 295 Cathedral Street, Glasgow G1 1XL, United Kingdom; b Wellcome Trust Centre for Molecular Parasitology, Institute of Infection, Immunity and Inflammation and Glasgow Polyomics, College of Medical, Veterinary and Life Sciences, University of Glasgow, Glasgow G12 8TA, United Kingdom; c Discovery Biology, Eskitis Institute for Drug Discovery, Griffith University, Nathan, Queensland 4111, Australia

**Keywords:** Minor Groove Binders, Antiparasitic activity, African trypanosomiasis

## Abstract

A series of 32 structurally diverse MGBs, derived from the natural product distamycin, was evaluated for activity against *Trypanosoma brucei brucei*. Four compounds have been found to possess significant activity, in the nanomolar range, and represent hits for further optimisation towards novel treatments for Human and Animal African Trypanosomiases. Moreover, SAR indicates that the head group linking moiety is a significant modulator of biological activity.

## Highlights

• Minor Groove Binders (MGBs) possess activity against *Trypanosma brucei brucei*.
• 5 MGBs have been identified as leads for future investigation, each with IC_50_ values lower than 40 nM.
• DNA remains a possible target for these MGBs in trypanosomes, similar to the case for bacteria.

## Graphical abstract

Intracellular localisation of fluorescent MGB in the nucleus and kinetoplast of *T*. *b*. *brucei*. N = nucleus; K = kinetoplast; arrow = other organelles. Bar = 10 μm.

**Figure:**
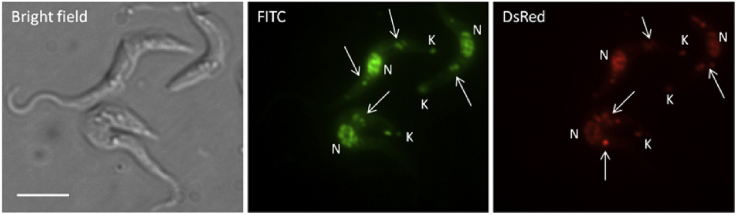

## 1 Introduction

In the last few decades there has been a significant increase in the amount of research directed at developing treatments for African trypanosomiasis and although few new drugs have emerged, notable progress has been made [1]. For example, 2009 saw the launch of a nifurtmox/eflornithine combination therapy for use against T. b. gambiense [2]. Drug design strategy towards African trypanosomiasis is currently undergoing a shift with an emphasis on new targets. *N*-Myristoyl transferase has recently been suggested as a potential molecular target for development after identification through target-based approaches [3]; however, a number of other similarly identified targets have not proven effective indicators of activity in *in vivo* experiments [4]. Consequently more effort has been placed on phenotypic screening and notable outcomes include oxaborole SCYX-7158, in phase I clinical trials, and fexinidazole, which is in phase III [5].

**Fig. 1:**
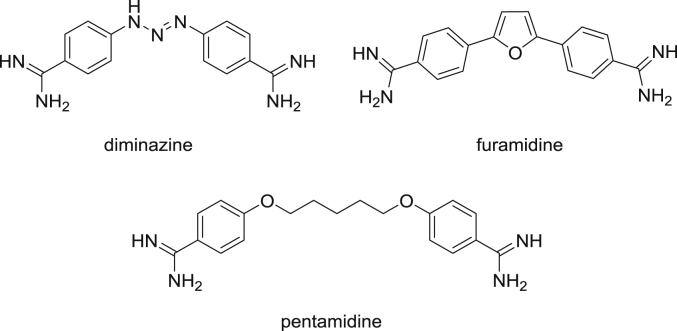
Bisamidine antitrypanosomal compounds.

Minor Groove Binders (MGBs) have been used against trypanosomiasis since the 1930s; in particular the bisamidines pentamidine and diminazene (Fig. 1) have been used as first-line treatments for Human African Trypanosomiasis (HAT) and Animal African Trypanosomiasis (AAT), respectively [6]. Many hundreds of related compounds have been synthesised and evaluated against trypanosomiasis and, additionally, many have found uses against protozoan infections, including leishmaniasis and malaria [7].

Although extensively investigated, the actual mechanism of action of MGBs in protozoa remains unclear and it is likely to differ in different species [8], [9]. It has been demonstrated that pentamidine, furamidine (Fig. 1), and a number of other diamidines are actively transported into both trypanosomes and plasmodia, achieving high intracellular concentrations. Moreover, these compounds bind strongly to AT-rich sites of the minor groove of parasitic DNA [8], [10]. Furamidine, for example, binds to an AT rich DNA oligonucleotide with K_D_ in the 100 nM range [10].

DNA binding as the proposed mechanism of action is strengthened considering that DNA binding correlates to some extent with trypanocidal activity [9], [10], [11]. The kinetoplast minicircles of trypanosomes are particularly AT-rich and thus present a likely target for diamidines. Furthermore, destruction of the kinetoplast has been observed within 24 h’ exposure to pentamidine and furamidine, leading to the death of the trypanosome [12] and in leishmania too loss of kinetoplast precedes death [13]. Although much effort continues towards the development of novel anti-protozoal diamidines, including clinical and preclinical trials [14], the problem of cross-resistance with current treatments remains. For example, mutations in the transporters involved in cellular drug accumulation have been implicated in pentamidine resistance [15].

**Fig. 2:**
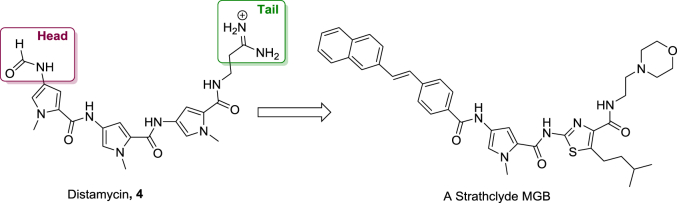
Distamycin and an example Strathclyde MGB.

At Strathclyde we have developed a collection of MGBs which are structurally based on the natural product distamycin. This compound is built from *N-*methylpyrrole amino acid amides and has an amidine end group (Fig. 2). In developing MGBs at Strathclyde, we modified a number of the regions of the prototype structure. Less basic functional groups have been introduced to replace the amidine at the C-terminus (usually referred to as the tail group because of its flexibility). Larger alkyl side chains have been substituted for methyl groups and thiazole rings have been introduced to the body of the MGB. Additionally, aromatic rings have replaced the formyl group from distamycin and the *N-*terminal amide has been replaced by its isosteric alkene [16], [17]. Although the structures of Strathclyde MGBs now differ substantially from the parent compound, distamycin, the preferential binding to AT-rich DNA, over GC, still remains for this class of compound in general [18].

In conjunction with our commercial partner, MGB Biopharma, we have had great success in developing our MGBs as antibacterials [18] and, crucially, structural features of these MGBs are quite distinct from the diamidines. For example, the terminal amidine groups that are critical to both activity and uptake of the diamidines are absent in these antibacterial distamycin analogues. Since loss of transport seems to underlie resistance to diamidines, it seems likely that cross–resistance to the Strathclyde MGBs would not occur should they be active. With new drugs a priority for both human and veterinary trypanosomiases, we have investigated our MGBs as potential antitrypanosomal agents. This study thus reveals our exploratory investigation and findings of Strathclyde MGB activity against *Trypanosoma brucei brucei* as a representative and relevant, initial target parasite. The results identify lead compounds with the potential for optimisation and development.

## 2 Results and discussion

### 2.1 Synthesis

**Fig. 3:**
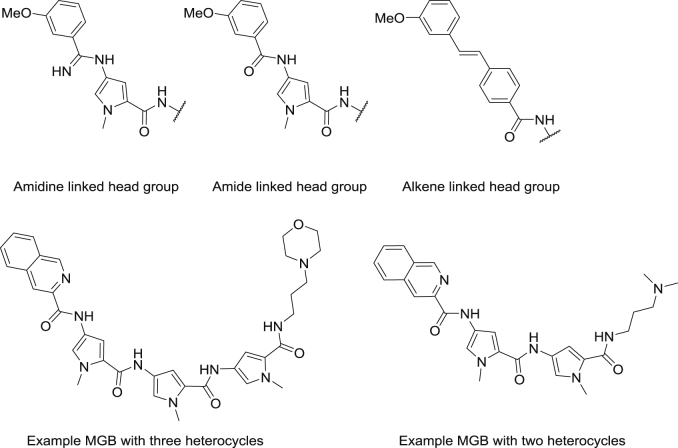
Exemplars of the types of MGB investigated in this study.

The MGBs investigated in this study possess two locations of diversity when considering their synthesis: the head group link and the number of heterocycles. The head group is linked by an amide, an amidine, or an alkene and the number of heterocycles between the head and the tail group is either two or three (Fig. 3). This set of compounds allows us to assess some key structural points with respect to the design of MGBs as antitrypanosomal agents.

**Scheme 1:**
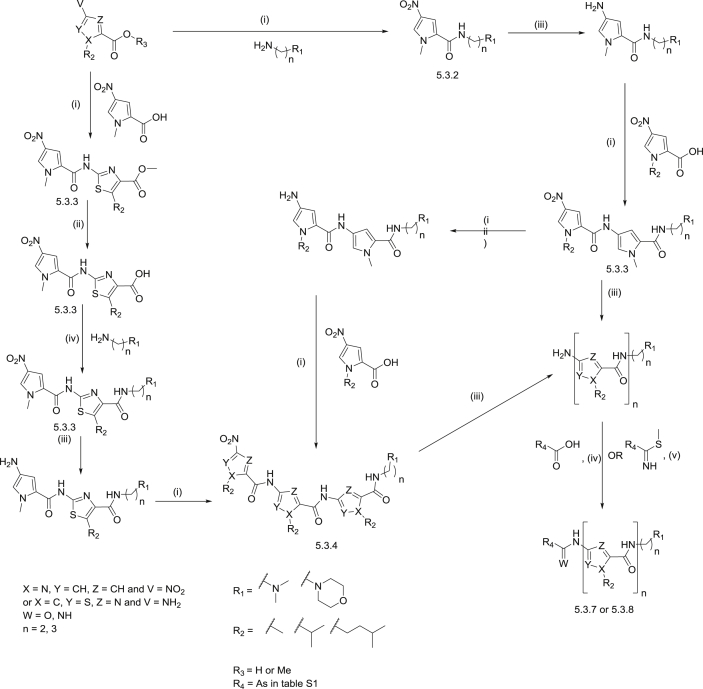
Synthetic strategy for MGBs. (i) Thionyl chloride, 50 °C, 4 h, then amine in DCM, rt, overnight, 43–60%; (ii) LiOH, MeOH:water (5:1), 48 h, 92–96% (iii) H_2_, Pd/C, MeOH, rt, 2 h, used immediately in next step; (iv) HBTU, DMF, rt, overnight, 95%; (v) DMF, rt, overnight, 14–64%. Compound numbers beneath structures refer to general synthetic methods in experimental section.

Synthesis of these MGBs (Scheme 1) was achieved in a modular fashion, initially building from the C-terminus, tail group end, of the molecule and ending with a coupling reaction to attach the head group. To build a thiazole-containing MGB, the acid chloride of the nitropyrrole carboxylic acid was made using thionyl chloride, followed by reacting with the methyl 2-amino-5-alkyl-1,3-thiazole-4-carboxylate. The methyl ester of the carboxylic acid was hydrolyzed using LiOH in water/methanol before an HBTU coupling was employed to attach the primary amine of the tail group to the carboxylic acid of the thiazole containing dimer. The nitro group of the N-terminus was reduced to an amine by hydrogenation using palladium over charcoal and a further pyrrole was attached by using thionyl chloride to prepare the corresponding acid chloride. For pyrrole-only containing MGBs, the tail group was attached to a pyrrole heterocyclic unit first. This was achieved by forming the acid chloride of the nitropyrrole carboxylic acid using thionyl chloride, followed by reacting with the appropriate tail group amine. Further pyrrole heterocyclic units were added as necessary using the same coupling method and reducing the nitro group to an amine as previously described. The final step to form the complete MGB depended on the particular link to the head group.

The final coupling to form the MGBs with an amide linked head group was carried out using HBTU. To do this, the nitro group of the appropriate tail group containing dimer or trimer was first reduced and reacted, without further purification, with the required head group carboxylic acid which had been charged with HBTU. Before the final coupling to form the alkene containing MGBs, the appropriate head group was first synthesised using a Horner–Wadsworth–Emmons reaction, as previously described [19]. As before, HBTU coupling was used to attach the head group to form the alkene containing MGB. Those MGBs with amidine linked head groups were prepared using a methyl imidothiolate head group. Briefly, the commercially available nitriles were converted into the corresponding thioamides by the use of thioacetamide. The thioamide was then reacted with methyl iodide in order to afford required methyl imidothiolate head groups. To form the amidine containing MGB the nitro group of the appropriate tail group containing trimer was reduced, as before, and reacted with one equivalent of the appropriate methyl imidothiolate head group.

All MGBs were purified by HPLC to give a final purity greater than 98%. Table S1 in the supplementary information shows the structures of the MGBs that were investigated in this study.

### 2.2 Biological activity

The majority of the MGBs investigated in this study display appreciable activity against *T*. *b*. *brucei* in the range of 0.1–2 μM (Table 1); however, close inspection of the structural types indicates that the amidine linked head group MGBs (**1**, **2**, **5**, **9**, **17**, **19**) are not strongly active. The most active of these, **17** and **19**, have an IC_50_ of 4.9 and 2.2 μM respectively, whilst the other amidine linked head group compounds, **1**, **2**, **5**, **9** all have IC_50_s greater than 75 μM. More evidence for the poor tolerance of a basic site in the head group end can be seen in comparing compounds **20** and **23**. Here, the removal of the basic site of the quinoline, through using the naphthalene, results in the IC_50_ decreasing from 0.62 μM to 40 nM.

The data also reveal that the set of shorter MGBs, **14**, **15** and **18**, retain activity against *T*. *b*. *brucei* and are comparable in activity to many of the longer MGBs. These compounds have IC_50_ values of 0.97, 2.2 and 0.39 μM respectively, which places them within the range of activity of most of the MGBs. This is an important point as the shorter length MGB could be anticipated to bind to DNA less strongly due to lower potential for stabilising van der Waals’ interactions.

A tentative conclusion can also be drawn in examining the effect of the tail group on activity by considering the compound pairs **14** and **15**, and **13** and **21**. These are matched pairs in which the molecules only differ by containing either morpholine or dimethylamine in the tail group. In both cases, the less basic morpholine tail group leads to greater activity as is particularly noticeable for compounds **13** and **21** where the activity dramatically increases from 0.21 μM to 5.4 nM.

Of great significance is the discovery of five compounds with IC_50_ values of less than 40 nM. Four of these compounds (**20**, **21**, **28** and **29)** belong to the alkene linked head group set, and the other (compound **8)** belongs to the amide linked head group set, The IC_50_ of the diamidine control, diminazene, in this assay is 5 nM and compounds **8**, **21** and **28** come particularly close to this with IC_50_ values of 6.8, 5.4 and 7.3 nM, respectively.

The five most active compounds were evaluated for their mammalian cytotoxicity using an AlamarBlue® viability assay using the human embryonic kidney cell line, HEK 293. These results are shown in Table 2.

Of these compounds, only **21** had a measureable cytotoxicity at the 20 μM test concentration. Furthermore, these five most active compounds all displayed selectivity indices at least greater than 500 indicating substantial parasite selectivity. From these data, **8** and **28** appear particularly attractive as candidates for lead optimisation due to their selectivity indices of >2941 and > 2739, respectively.

### 2.3 In silico physicochemical properties prediction

**Fig. 4:**
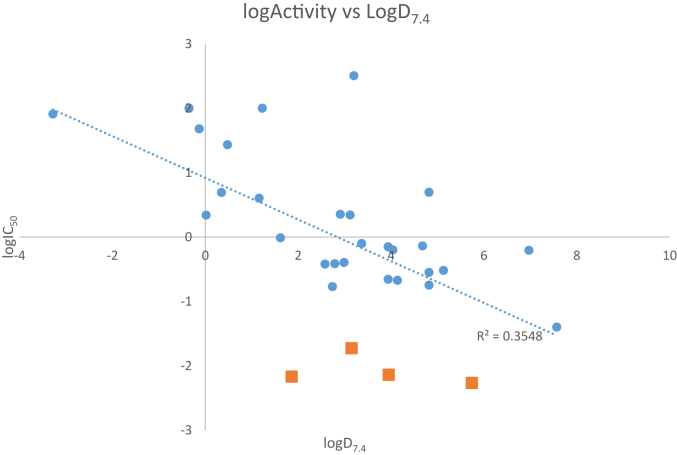
LogD_7.4_ against MGB activity. Orange squares indicate the most active compounds. (For interpretation of the references to colour in this figure legend, the reader is referred to the web version of this article.)

A number of standard physicochemical properties were predicted using the software MarvinSketch (Version 15.6.29.0, ChemAxon, http://www.chemaxon.com), in an attempt to gain some insight into the differences in activity. Full details of these can be found in the supplementary electronic information; however, a representative selection is presented here for discussion. Fig. 4 depicts the relationship between the calculated logD_7.4_ and the log of the activity, which shows a weak negative correlation between the two. This relationship holds for logD estimates at pHs 6.5, 5.0 and 1.5 also (see supplementary information).

**Fig. 5:**
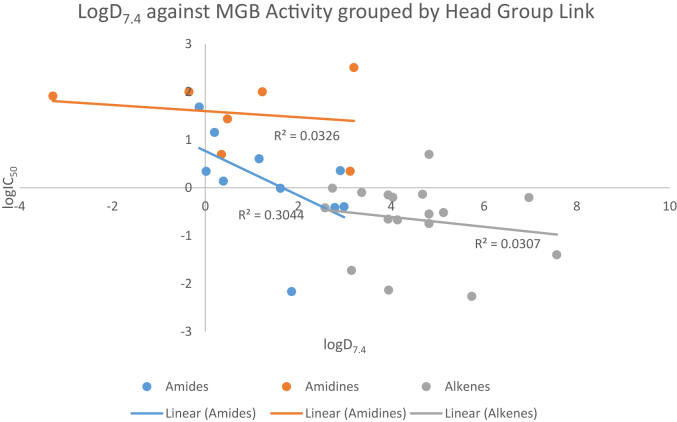
LogD_7.4_ against MGB activity grouped by head group link.

Fig. 5 shows the data grouped by head group type, with separate correlation coefficients, and suggests that the three head group types have different characteristics with respect to this measure of correlation. Moreover, the four most active compounds appear to be outliers (orange squares in Fig. 4) suggesting that some specific interactions with a component of the target organism might be responsible for the high activity.

It is possible that specific biological transporters are important in determining the activity of these compounds rather than passive transport across membranes. A specific example of this would be in the comparison of compounds **27** and **28** which have IC_50_ values of 0.22 μM and 7.3 nM, respectively. Both of these compounds have the same predicted values of logD across pHs 1.5, 5.0, 6.5 and 7.4 and so would be expected to have the same membrane permeabilities, yet their activities significantly differ. Similarly, the lack of activity for most of the amidine linked head group MGBs may be due to a specific biological event, such as increased cellular efflux (or decreased influx).

Compound **8** (IC_50_ = 6.8; logD_7.4_ = 1.9) would appear from the above analysis to be the most attractive compound for medicinal chemical optimisation. LogD_7.4_ with this template could be lowered by increasing the pK_a_ of the tail group or by introducing a more polar head group such as those of the active alkenes, **28** and **29**. The least lipophilic active alkene, **29** (IC_50_ = 19 nM; logD_7.4_ = 3.2), is also in a suitable property range to be a lead for optimisation. Such optimisation in the next stage of the project will necessarily involve consideration of pharmokinetic, metabolic, and toxicity properties but the intrinsic flexibility of the Strathclyde MGB template allows for great structural variety to be introduced.

### 2.4 Fluorescence microscopy

**Fig. 6:**
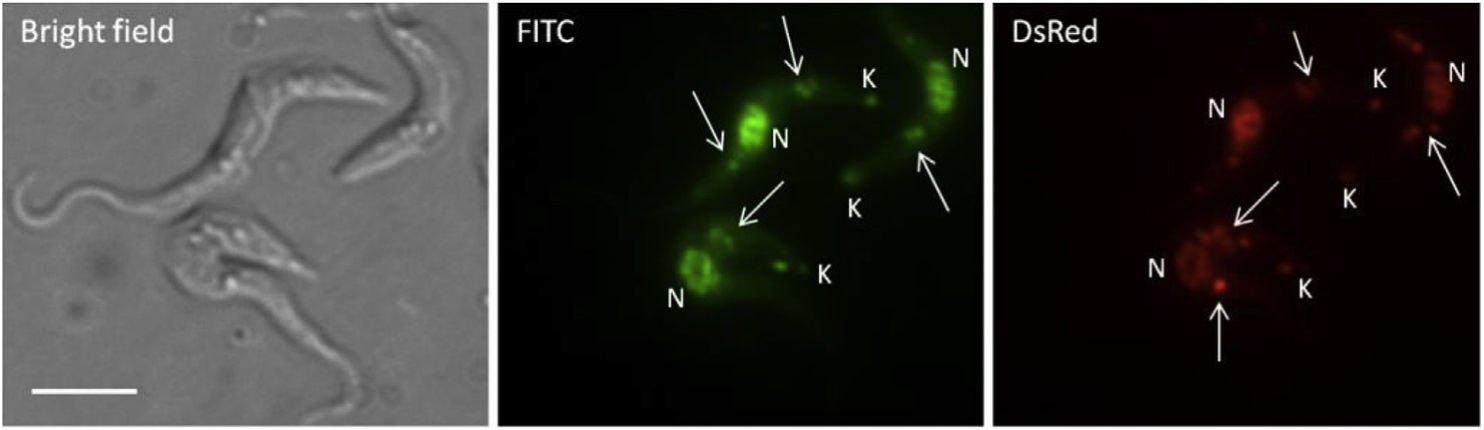
Intracellular localisation of fluorescent MGB in *T*. *b*. *brucei*. Trypanosomes were treated for 2 h with 50 μM of MGB and viewed at 100 × magnification. N = nucleus; K = kinetoplast; arrow = other organelles. Bar = 10 μm.

To investigate DNA binding as a plausible mechanism of action for this series of MGBs, compound **32**, which is strongly fluorescent although not highly active, was used to study intracellular localisation by fluorescence microscopy (Fig. 6). This experiment confirmed internalisation of compound **32** into the trypanosome, where localisation to the nucleic acid rich regions of the nucleus and kinetoplast was observed. In addition to these, a punctate staining pattern of unknown origin was visible under both FITC and DsRed filters.

The localisation of an MGB in organelles other than those rich in nucleic acids is of interest to understand their mechanism of action. In the field of MGBs as antibacterials, it has been suggested that DNA binding may not be the only mechanism leading to antibacterial activity [20]. However, although other cellular targets may exist, interference with DNA centric events are still thought to be the most mechanism of action of these MGBs against trypanosomes.

## 3 Conclusion

In this study we have demonstrated that our MGBs possess activity against *T*. *b*. *brucei*. Furthermore, five compounds have been identified as leads for future investigation, each with IC_50_ values lower than 40 nM. The spectrum of activities that have been observed would suggest that specific biological transporters are involved in MGB uptake and not only passive transport across membranes – the head group link being of principal importance.

Designing selective antimicrobials is of importance in attempting to curtail the increase in antimicrobial resistance and this study has also contributed some new knowledge to our antibacterial MGB design. All the MGBs in this study are known to possess a range of antibacterial activity [19, unpublished data]; however, this study has revealed that focusing our investigation on those with amidine linked head groups may allow the development of antibacterials without antitrypanosomal activity.

Furthermore, we have demonstrated that DNA remains a possible target for these MGBs in trypanosomes similar to the case for bacteria.

## 4 Experimental

### 4.1 Biological activity

#### 4.1.1 Trypanosoma brucei brucei assay

Bloodstream form *T*. *b*. *brucei* (Lister 427) was cultivated in HMI-9 medium (Gibco) supplemented with 10% heat inactivated FBS (Gibco), at 37 °C in a humidified 5% CO_2_ environment. IC_50_ values against trypanosomes were determined by Alamar Blue assay. Briefly, 2 × 10^4^ cells/mL were seeded into serial dilutions of the test compounds and incubated for 48 h at 37 °C, 5% CO_2_. After this time, 20 μL of resazurin dye (Sigma–Aldrich) solution at 0.49 mM was added. Following further 24 h incubation the reduction of resazurin was measured using a fluorimeter (FLUOstar Optima, BMG Labtech) at 544 nm excitation and 590 nm emission wavelengths. Output was plotted using the IC_50_ determination algorithm of Prism 5.0 software (GraphPad). Three independent experiments were carried out in duplicate for each compound.

#### 4.1.2 HEK 293 cytotoxicity assay

Compounds were tested in an AlamarBlue viability assay using fluorescent intensity as the output. Briefly, Human Embryonic Kidney cells (HEK 293) were cultured in DMEM (Dulbeccos Modified Eagle Medium) plus 10% Foetal Bovine Serum (FBS) in a humidified environment at 37 °C and 5% CO_2_. Cells were harvested at 80% confluence and plated into 384-well tissue culture treated black walled clear base plates at 2000 cells per well in a volume of 45 μl and incubated overnight to equilibrate. Five μl of compounds in serial dilution format, including Puromycin as a positive control and 0.4% DMSO as the negative control, were transferred into the plates containing the equilibrated HEK293 cells. The plates were incubated for a further 72 h before “flicking” the compound containing media out and replacing with 40 μL of 10% AlamarBlue® in DMEM without FBS. The assay plates were returned to the incubator for 6 h then the total fluorescent intensity determined at 535 nm excitation and 585 nm emission using the Perkin Elmer ENVISION 2101 Multilabel Reader. Fluorescent intensity values were normalized as percent inhibition in relation to the positive and negative controls of 5 μM Puromycin and 0.4% DMSO, respectively. The compounds were tested in two separate experiments each in duplicate point.

### 4.2 Fluorescence microscopy

*T*. *b*. *brucei* parasites (1 × 10^7^ cell/mL) were treated with 50 μM of compound **32** for 2 h at 37 °C in a humidified, 5% CO_2_ atmosphere. After washing in PBS, an aliquot of cells was mixed with an equal volume of 2% w/v low melting point agarose (Sigma–Aldrich) and spread onto a slide by addition of a coverslip. Slides were viewed using a Zeiss Axioplan 2 Imaging epifluorescence microscope, equipped with a digital CCD camera (Hammatsu Photonics) and a PlanNEUFLUAR 100 × oil-immersion NA 1.4 objective (Carl Zeiss). FITC (excitation 450–490 nm, beamsplitter 510 nm, emission 515–565 nm) and DsRed (excitation 545/25 nm, beamsplitter 570 nm, emission 605/70 nm) filter sets were used, and images acquired using Volocity imaging software (Improvision).

### 4.3 Synthesis

#### 4.3.1 General experimental methods

^1^H and ^13^C NMR spectra were measured on a Bruker DPX-400 MHz spectrometer with chemical shifts given in ppm (δ-values), relative to proton and carbon traces in solvent. Coupling constants are reported in Hz. IR spectra were recorded on a Perkin Elmer, 1 FT-IR spectrometer. Mass spectra were obtained on a Jeol JMS AX505. Anhydrous solvents were obtained from a Puresolv purification system, from Innovative Technologies, or purchased as such from Aldrich. Melting points were recorded on a Reichert hot-stage microscope, and are uncorrected. Chromatography was carried out using 200–400 mesh silica gels, or using reverse-phase HPLC on a waters system using a C18 Luna column.

#### 4.3.2 General synthesis of pyrrole monomer with tail group

1-Methyl-4-nitro-1*H*-pyrrole-2-carboxylic acid (500 mg, 2.94 mmol) was suspended in thionyl chloride (5 mL) then the reaction mixture was heated under reflux for 4 h. Excess thionyl chloride was removed under reduced pressure at 50 °C and the acid chloride so formed was dissolved in DCM (5 mL, dry) and trimethylamine (2.94 mmol) was added. To this was added the appropriate primary amine containing tail group (2.94 mmol) in DCM (1 mL). The addition was dropwise with stirring and the stirring was continued overnight. The solvent was removed under reduced pressure and the crude product was purified by column chromatography using methanol/ethyl acetate (1/4) containing 1% TEA. Fractions containing the product were collected and the solvents were removed under reduced pressure to give the pure product.

#### 4.3.3 General synthesis of dimers

##### 4.3.3.1 Thiazole methyl ester dimer

1-Methyl-4-nitro-1*H*-pyrrole-2-carboxylic acid (500 mg, 2.94 mmol) was suspended in thionyl chloride (5 mL) then the reaction mixture was heated under reflux for 4 h. Excess thionyl chloride was removed under reduced pressure at 50 °C and the acid chloride so formed was dissolved in DCM (5 mL, dry). The appropriate 2-aminothiazole methyl ester (3.19 mmol) was dissolved in DCM (5 mL, dry) to which triethylamine (0.5 mL, dry) was added with stirring at room temperature. The acid chloride solution was added to the amine solution dropwise with stirring at room temperature and the stirring was continued overnight. The reaction mixture was extracted with KOH solution (840 mg, in water 10 mL). The organic layer was extracted with brine, dried (MgSO_4_) and the solvent removed under reduced pressure. The crude product was applied to a column chromatography using silica gel and ethyl acetate/n-hexane as eluant (1/4) to afford the desired product.

##### 4.3.3.2 Thiazole carboxylic acid dimer

The thiazole methyl ester dimer (1.74 mmol) was suspended in a mixture of water (25 mL) and methanol (5 mL) containing lithium hydroxide (125 mg, 5.21 mmol). The reaction mixture was stirred vigorously for 48 h at room temperature. Some of the methanol was removed under partial reduced pressure at 50 °C. The cooled solution was extracted with ether and the ethereal layer was discarded. The aqueous layer was cooled to 0 °C and acidified by adding HCl (Concentrated) dropwise with stirring. The pale yellow solid was filtered off, washed with water and dried overnight at 45 °C under reduced pressure to give the required material.

##### 4.3.3.3 Thiazole containing dimer with tail group

The thiazole carboxylic acid dimer (1.56 mmol) was dissolved in DMF (2.5 mL, dry) to which thiethylamine (0.5 mL, dry), HBTU (1.18 g, 3.12 mmol) and the appropriate primary amine containing tail group (3.12 mmol) were added at room temperature with stirring. Ethyl acetate and dilute sodium hydroxide solution were added. After the extraction, the organic layer was collected, dried (Na_2_SO_4_), filtered and the solvent was removed under reduced pressure. The crude product was applied to a silica gel column and eluted with ethyl acetate/methanol (1/1) containing 1% TEA. Fractions containing the required product were collected and the solvents were removed under reduced pressure to give the desired product.

##### 4.3.3.4 Pyrrole-only containing dimer with tail group

The appropriate pyrrole monomer with tail group (1.06 mmol) was dissolved in methanol (25 mL) to which Pd/C-10% (135 mg) was added. The reaction mixture was hydrogenated for 3 h at room temperature and atmospheric pressure. The catalyst was removed over Kieselguhr and the solvent was removed under reduced pressure. The amine so formed was dissolved in DCM (2 mL, dry), to which triethylamine (0.3 mL) was added. 1-Methyl-4-nitro-1*H*-pyrrole-2-carboxylic acid (181 mg, 1.06 mmol) was suspended in thionyl chloride (2 mL), and heated under reflux for 2 h. Excess thionyl chloride was removed under reduced pressure to give the acid chloride, which was dissolved in DCM (2 mL, dry). The acid chloride solution was added dropwise to the amine solution at room temperature with stirring. The stirring was continued overnight. The reaction mixture was extracted with potassium hydroxide solution (KOH 588 mg in water 10 mL). The organic layer was dried (MgSO_4_), the solved was removed under reduced pressure and the crude product was purified by column chromatography, methanol/ethyl acetate (1/4) containing 1% TEA to give the required product.

#### 4.3.4 General synthesis of trimer

The appropriate dimer (0.65 mmol) was dissolved in methanol (25 mL) to which Pd/C-10% (150 mg) was added. The reaction mixture was hydrogenated for 4 h at room temperature and atmospheric pressure. The catalyst was removed over Kieselguhr and the solvent was removed under reduced pressure. The amine so formed was dissolved in DCM (2 mL, dry), to which triethylamine (0.3 mL) was added. 1-Methyl-4-nitro-1*H*-pyrrole-2-carboxylic acid (0.65 mmol) was suspended in thionyl chloride (2 mL), and heated under reflux for 2 h. Excess thionyl chloride was removed under reduced pressure to give the acid chloride, which was dissolved in DCM (2 mL, dry). The acid chloride solution was added dropwise to the amine solution at room temperature with stirring. The stirring was continued overnight. The reaction mixture was extracted with potassium hydroxide solution (KOH 588 mg in water 10 mL). The organic layer was dried (MgSO_4_), the solved was removed under reduced pressure and the crude product was purified by column chromatography using methanol/ethyl acetate (1:5) containing 1% TEA to give the required product.

#### 4.3.5 General synthesis of alkene head group

##### 4.3.5.1 Head group methyl ester

The appropriate diethylphosphonate (1.15 mmol) was dissolved in THF (5 mL, dry) then cooled to 0 °C under nitrogen. Sodium hydride (92 mg, 60% suspension in oil, 2.30 mmol) was added portionwise with stirring. The appropriate aldehyde (1.15 mmol) was dissolved in THF (5 mL, dry) then added to the reaction mixture at 0 °C under nitrogen with stirring. The reaction mixture was then left stirring at room temperature for 1 h. Water was added at 0 °C, dropwise, under nitrogen followed by dilute HCl until pH 6. The reaction mixture was diluted with ethyl acetate and extracted. The water layer was extracted once more with ethyl acetate and the combined organic layers were dried (MgSO_4_), filtered and the solvents were removed under reduced pressure. The product was purified by column chromatography using ethyl acetate/n-hexane (1:2) to give the required methyl ester of the head group.

##### 4.3.5.2 Head group carboxylic acid

The methyl ester of the head group (0.32 mmol) was dissolved in methanol (5 mL) to which sodium hydroxide solution (NaOH 200 mg, 5 mmol in water 10 mL) was added. The reaction mixture was heated under reflux for 3 h. Methanol was removed under partial reduced pressure and the remaining solution was cooled to 0 °C. Hydrochloric acid (conc.) was added dropwise with vigorous stirring until pH 4. The material was collected by filtration, washed with water and dried under reduced pressure at 50 °C to give the required material.

#### 4.3.6 General synthesis of head group for amidine linked MGB

The appropriate thioamide (0.55 mmol) was suspended in acetone (25 mL, dry) to which iodomethane (200 μL) was added and the reaction mixture was heated under reflux for 6 h and the product was filtered off, washed with small amount of dry acetone and dried under reduced pressure at 45 °C overnight to give the required product.

#### 4.3.7 General synthesis of alkene or amide MGB

The tail group containing dimer or trimer (0.266 mmol) was dissolved in methanol (25 mL), to which Pd/C-10% (60 mg) was added at 0 °C under nitrogen with stirring. The reaction mixture was hydrogenated at room temperature and atmospheric pressure for 4 h. The catalyst was removed over Kieselguhr and the solvent was removed under reduced pressure to give the amine, which was dissolved in DMF (1 mL, dry). The appropriate head group carboxylic acid (0.266 mmol) was dissolved in DMF (1 mL, dry) to which HBTU (260 mg, 0.685 mmol) and triethylamine (50 μL) were added to the reaction mixture at room temperature with stirring and the reaction mixture was left standing at room temperature overnight. The product was purified by HPLC (no work up required). Fractions containing the product were collected and freeze dried to give the required product.

#### 4.3.8 General synthesis of amidine linked MGB

The tail group containing trimer (0.401 mmol) was dissolved in methanol (25 mL), cooled to 0 °C then Pd/C-10% (135 mg) was added with stirring. The reaction mixture was hydrogenated for 4 h at room temperature and atmospheric pressure. The catalyst was removed over Kieselguhr and to this was added the hydrogen iodide salt of the appropriate methyl imidothiolate (0.26 mmol) at room temperature with stirring, and then the reaction mixture was kept at room temperature for 48 h. Solvent was removed under reduced pressure and the product was purified by HPLC (no work up required). Fractions containing the product were collected and freeze dried to give the required product.

#### 4.3.9 *N*-[5-({[5-({[3-(Dimethylamino)propyl]amino}carbonyl)-1-methyl-1*H*-pyrrol-3-yl]amino}carbonyl)-1-methyl-1*H*-pyrrol-3-yl]-4-{[imino(phenyl)methyl]amino}-1-methyl-1*H*-pyrrole-2-carboxamide **1**

Prepared as per [19].

#### 4.3.10 N-[5-({[5-({[3-(Dimethylamino)propyl]amino}carbonyl)-1-methyl-1H-pyrrol-3-yl]amino}carbonyl)-1-methyl-1H-pyrrol-3-yl]-4-(ethanimidoylamino)-1-methyl-1H-pyrrole-2-carboxamide **2**

Prepared as per [19].

#### 4.3.11 4-[(2,4-Dichlorobenzoyl)amino]-1-methyl-N-[1-methyl-5-({[1-methyl-5-({[2-(4-morpholinyl)ethyl]amino}carbonyl)-1H-pyrrol-3-yl]amino}carbonyl)-1H-pyrrol-3-yl]-1H-pyrrole-2-carboxamide **3** (synthesis also reported in [23])

White solid (34 mg, 41%) with no distinct melting point. ^1^H NMR (DMSO-d_6_): 10.48 (1H, s), 9.94 (1H, s), 9.91 (1H, s), 9.53 (1H, br), 8.21 (1H, t, *J* = 5.5 Hz), 7.74 (1H, d, *J* = 1.7 Hz), 7.55–7.53 (2H, m), 7.26 (1H, d, *J* = 1.7 Hz), 7.23 (1H, d, *J* = 1.7 Hz), 7.19 (1H, d, *J* = 1.7 Hz), 7.07 (1H, d, *J* = 1.7 Hz), 7.02 (1H, d, *J* = 1.7 Hz), 7.00 (1H, d, *J* = 1.7 Hz), 3.99 (2H, m), 3.87 (3H, s), 3.85 (3H, s), 3.82 (3H, s), 3.53–2.99 (10H, m). IR (KBr): 1665, 1550, 1290, 1200, 1131 cm^−1^. HRFABMS: found: 669.2112 calculated for C_31_H_35_Cl_2_N_8_O_5_ 669.2107.

#### 4.3.12 4-(Formylamino)-1-methyl-*N*-[1-methyl-5-({[1-methyl-5-({[2-(4-morpholinyl)ethyl]amino}carbonyl)-1*H*-pyrrol-3-yl]amino}carbonyl)-1*H*-pyrrol-3-yl]-1*H*-pyrrole-2-carboxamide **4**

Brown solid (26 mg, 38%) with no distinct melting point. ^1^H NMR (DMSO-d_6_): 10.02 (1H, s), 9.91 (1H, s), 9.89 (1H, s), 9.52 (1H, br), 8.20 (1H, t, *J* = 5.5 Hz), 8.13 (1H, d, *J* = 1.7 Hz), 7.21 (1H, d, *J* = 1.7 Hz), 7.19 (1H, s), 7.18 (1H, d, *J* = 1.7 Hz), 7.06 (1H, d, *J* = 1.7 Hz), 7.00 (1H, d, *J* = 1.7 Hz), 6.92 (1H, d, *J* = 1.7 Hz), 4.02–3.99 (2H, m), 3.847 (3H, s), 3.845 (3H, s), 3.82 (3H, s), 3.72–3.13 (10H, m). IR (KBr): 1665, 1550, 1290, 1200, 1131 cm^−1^. HRFABMS: found: 525.2565 calculated for C_25_H_33_N_8_O_5_ 525.2574.

#### 4.3.13 2-{[(4-{[(4-{[Imino(4-methoxy-2-pyridinyl)methyl]amino}-1-methyl-1*H*-pyrrol-2-yl)carbonyl]amino}-1-methyl-1*H*-pyrrol-2-yl)carbonyl]amino}-5-isopropyl-*N*-[2-(4-morpholinyl)ethyl]-1,3-thiazole-4-carboxamide **5**

White solid (20 mg, 32%) with no distinct melting point. ^1^H NMR (DMSO-d_6_): 12.05 (1H, s), 11.12 (1H, s), 10.11 (1H, s), 9.78 (2H, s & br), 8.83 (1H, s), 8.08 (1H, br), 7.59 (1H, t, *J* = 7.9 Hz), 7.46–7.29 (6H, m), 7.08 (1H, d, *J* = 1.7 Hz), 4.20 (1H, septet, *J* = 6.9 Hz), 4.10 (2H, m), 3.95 (3H, s), 3.90 (3H, s), 3.88 (3H, s), 365–3.12 (10H, m), 1.29 (6H, d, *J* = 6.9 Hz). IR (KBr): 1665, 1549, 1273, 1200, 1134 cm^−1^.HRFABMS: found: 676.3032 calculated for C_33_H_42_O_5_N_9_S 676.3030.

#### 4.3.14 5,6-Dichloro-*N*-[5-({[5-({[5-({[3-(dimethylamino)propyl]amino}carbonyl)-1-methyl-1*H*-pyrrol-3-yl]amino}carbonyl)-1-isopropyl-1*H*-pyrrol-3-yl]amino}carbonyl)-1-methyl-1*H*-pyrrol-3-yl]nicotinamide **6**

*P*ale yellow solid (51 mg, 46%) after freeze-drying. ^1^H NMR (DMSO-d_6_): 10.67 (1H, s), 10.00 (1H, s), 9.93 (1H, s), 9.26 (1H, br), 8.89 (1H, d, *J* = 2.1 Hz), 8.59 (1H, d, *J* = 2.1 Hz), 8.14 (1H, t, *J* = 6.4 Hz), 7.42 (1H, d, *J* = 1.7 Hz), 7.34 (1H, d, *J* = 1.4 Hz), 7.17 (1H, d, *J* = 1.4 Hz), 7.11 (1H, d, *J* = 1.7 Hz), 5.98 (1H, d, *J* = 1.7 Hz), 8.95 (1H, d, *J* = 1.7 Hz), 5.44 (1H, quintet, *J* = 6.7 Hz), 3.89 (3H, s), 3.81 (3H, s), 3.25 (2H, q, *J* = 6.1 Hz), 3.07 (2H, m), 2.79 (6H, d, *J* = 4.1 Hz), 1.84 (2H, quintet, *J* = 8.0 Hz), 1.38 (6H, d, *J* = 6.7 Hz). IR (KBr): 1650, 1582, 1529, 1439, 1406, 1250, 1200, 1131 cm^−1^. HRFABMS: found: 670.2483 calculated for C_31_H_38_N_9_O_4_^35^Cl_2_ 670.2423.

#### 4.3.15 *N*-[5-({[5-({[3-(Dimethylamino)propyl]amino}carbonyl)-1-methyl-1*H*-pyrrol-3-yl]amino}carbonyl)-1-isopropyl-1*H*-pyrrol-3-yl]-4-{[imino(3-methoxyphenyl)methyl]amino}-1-methyl-1*H*-pyrrole-2-carboxamide**7**

Prepared as per [19].

#### 4.3.16 *N*-[1-Methyl-5-({[1-methyl-5-({[1-methyl-5-({[3-(4-morpholinyl)propyl]amino}carbonyl)-1*H*-pyrrol-3-yl]amino}carbonyl)-1*H*-pyrrol-3-yl]amino}carbonyl)-1*H*-pyrrol-3-yl]-3-isoquinolinecarboxamide **8** (synthesis also reported in [21])

Yellow solid (47 mg, 55%) with no distinct melting point (after the purification by HPLC). ^1^H NMR (DMSO-d_6_): 10.81 (1H, s), 9.98 (1H, s), 9.91 (1H, s), 9.64 (1H, br), 9.45 (1H, s), 8.64 (1H, s), 8.29 (1H, d, *J* = 8.0 Hz), 8.24 (1H, d, *J* = 8.0 Hz), 8.16 (1H, t, *J* = 6.0 Hz), 7.92 (1H, dt, *J* = 1.2 Hz & *J* = 7.2 Hz), 7.83 (1H, dt, *J* = 1.2 Hz & *J* = 7.2 Hz), 7.44 (1H, d, *J* = 1.7 Hz), 7.30 (1H, d, *J* = 1.7 Hz), 7.25 (1H, d, *J* = 1.7 Hz), 7.18 (1H, d, *J* = 1.7 Hz), 7.09 (1H, d, *J* = 1.7 Hz), 6.96 (1H, d, *J* = 1.7 Hz), 4.05–4.00 (2H, m), 3.91 (3H, s), 3.87 (3H, s), 3.82 (3H, s), 3.75–3.06 (10H, m), 1.88 (2H, quintet, J = 6.8 Hz). IR (KBr): 1665, 1550, 1290, 1200, 1131 cm^−1^. HRFABMS: found: 666.3173 calculated for C_35_H_40_N_9_O_5_ 666.3152.

#### 4.3.17 4-{[(4-{[imino(3-methoxyphenyl)methyl]amino}-1-methyl-1*H*-pyrrol-2-yl)carbonyl]amino}-1-methyl-*N*-[1-methyl-5-({[3-(4-morpholinyl)propyl]amino}carbonyl)-1*H*-pyrrol-3-yl]-1*H*-pyrrole-2-carboxamide **9**

Prepared as per [19].

#### 4.3.18 *N*-[3-(Dimethylamino)propyl]-5-isopentyl-2-({[1-methyl-4-({4-[(*E*)-2-(3-quinolinyl)ethenyl]benzoyl}amino)-1*H*-pyrrol-2-yl]carbonyl}amino)-1,3-thiazole-4-carboxamide **10**

Prepared as per [19].

#### 4.3.19 *N*-[3-(dimethylamino)propyl]-5-isopentyl-2-({[4-({4-[(*E*)-2-(3-methoxyphenyl)ethenyl]benzoyl}amino)-1-methyl-1*H*-pyrrol-2-yl]carbonyl}amino)-1,3-thiazole-4-carboxamide **11**

Prepared as per [19].

#### 4.3.20 N-[3-(Dimethylamino)propyl]-5-isopentyl-2-({[4-({3-[(E)-2-(3-methoxyphenyl)ethenyl]benzoyl}amino)-1-methyl-1H-pyrrol-2-yl]carbonyl}amino)-1,3-thiazole-4-carboxamide **12**

White solid (26 mg, 28%), with no distinct melting point. ^1^H NMR (DMSO-d_6_): 12.13 (1H, s), 10.48 (1H, s), 9.51 (1H, br), 8.15 (1H, br), 7.96 (1H, t, *J* = 6.1 Hz), 7.84 (2H, q, *J* = 7.8 Hz), 7.55 (2H, m), 7.45 (1H, d, *J* = 1.7 Hz), 7.37–7.29 (3H, m), 7.23–7.21 (2H, m), 6.89–6.87 (1H, m), 3.93 (3H, s), 3.81 (3H, s), 3.36 (2H, t, *J* = 6.5 Hz), 3.22 (2H, t, *J* = 7.7 Hz), 3.11–3.05 (2H, m), 2.79 (6H, d, *J* = 4.8 Hz), 1.91(2H, t, *J* = 7.7 Hz), 1.63–1.50 (3H, m), 0.93 (6H, d, *J* = 6.4 Hz). IR (KBr): IR (KBr): 1682, 1641, 1583, 1540, 1464, 1435, 1404, 1266, 1202, 1134 cm^−1^. HRFABMS: Found: 657.3226 Calculated for C_36_H_45_O_4_N_6_S 657.3218.

#### 4.3.21 N-[3-(dimethylamino)propyl]-5-isopentyl-2-[({1-methyl-4-[({1-methyl-4-[(E)-2-(4-nitrophenyl)ethenyl]-1H-pyrrol-2-yl}carbonyl)amino]-1H-pyrrol-2-yl}carbonyl)amino]-1,3-thiazole-4-carboxamide **13**

Orange solid (17 mg, 18%), with no distinct melting point. ^1^H NMR (DMSO-d_6_): 12.09 (1H, s), 10.15 (1H, s), 9.41 (1H, br), 8.19 (2H, d, *J* = 8.9 Hz), 7.99 (1H, t, *J* = 6.1 Hz), 7.76 (2H, d, *J* = 8.9 Hz), 7.46 (1H, d, *J* = 1.7 Hz), 7.41 (2H, dd, *J* = 1.7 Hz & d, *J* = 16.2 Hz), 7.33 (1H, d, *J* = 1.7 Hz), 7.27 (1H, d, *J* = 1.7 Hz), 6.96 (1H, d, *J* = 16.2 Hz), 3.90 (6H, s), 3.36 (2H, q, *J* = 6.5 Hz), 3.21 (2H, t, *J* = 7.7 Hz), 3.11–3.06 (2H, m), 2.79 (6H, d, *J* = 4.8 Hz), 1.91 (2H, t, *J* = 6.8 Hz), 1.63–1.49 (3H, m), 0.93 (6H, d, *J* = 6.4 Hz). IR (KBr): 1680, 1645, 1580, 1544, 1461, 1440, 1414, 1269, 1206, 1133 cm^−1^. HRFABMS: Found: 675.3082 Calculated for C_34_H_43_O_5_N_8_S 675.3072.

#### 4.3.22 *N*-[1-Methyl-5-({[1-methyl-5-({[2-(4-morpholinyl)ethyl]amino}carbonyl)-1*H*-pyrrol-3-yl]amino}carbonyl)-1*H*-pyrrol-3-yl]-3-isoquinolinecarboxamide **14**

Yellow solid (85 mg, 54%) with no distinct melting point. ^1^H NMR (DMSO-d_6_): 10.81 (1H, s), 9.98 (1H, s), 9.63 (1H, s), 9.44 (1H, br), 8.63 (1H, s), 8.29 (1H, d, *J* = 8.0 Hz), 8.24 (1H, d, *J* = 8.0 Hz overlapped with 1H broad), 7.92 (1H, t, *J* = 7.2 Hz), 7.85 (1H, t, *J* = 7.2 Hz), 7.43 (1H, d, *J* = 1.7 Hz), 7.30 (1H, d, *J* = 1.7 Hz), 7.22 (1H, d, *J* = 1.7 Hz), 7.02 (1H, d, *J* = 1.7 Hz), 4.03 (2H, m), 3.88 (3H, s), 3.83 (3H, s), 3.67 (2H, t, *J* = 12.0 Hz), 3.05 (4H, m), 3.28 (2H, m), 3.13 (2H, m). IR (KBr): 1672, 1584, 1539, 1463, 1436, 1405, 1268, 1201, 1136 cm^−1^. HRFABMS: found: 530.2515 calculated for C_28_H_32_O_4_N_7_ 530.2516.

#### 4.3.23 *N*-[5-({[5-({[3-(Dimethylamino)propyl]amino}carbonyl)-1-methyl-1*H*-pyrrol-3-yl]amino}carbonyl)-1-methyl-1*H*-pyrrol-3-yl]-3-isoquinolinecarboxamide **15**

Yellow solid (88 mg, 54%) with no distinct melting point. ^1^H NMR (DMSO-d_6_): 10.80 (1H, s), 9.95 (1H, s), 9.44 (1H, s), 8.63 (1H, s), 8.29 (1H, d, *J* = 8.0 Hz), 8.23 (1H, d, *J* = 8.0 Hz), 8.15 (1H, t, *J* = 5.6 Hz), 7.91 (1H, t, *J* = 7.2 Hz), 7.83 (1H, t, *J* = 7.2 Hz), 7.43 (1H, s), 7.28 (1H, s), 7.19 (1H, s), 6.95 (1H, s), 3.85 (3H, s), 3.82 (3H, s), 3.26 (2H, q, *J* = 6.0 Hz), 3.08 (2H, m), 2.80 (6H, d, *J* = 4.8 Hz), 1.85 (2H, quintet, *J* = 8.0 Hz). IR (KBr): 1674, 1584, 1538, 1469, 1437, 1405, 1267, 1200, 1140 cm^−1^. HRFABMS: found: 502.2566 calculated for C_27_H_32_O_3_N_7_ 502.2567.

#### 4.3.24 4-({4-[(*E*)-2-(1,3-benzoxazol-2-yl)ethenyl]benzoyl}amino)-1-methyl-*N*-[1-methyl-5-({[2-(4-morpholinyl)ethyl]amino}carbonyl)-1*H*-pyrrol-3-yl]-1*H*-pyrrole-2-carboxamide **16**

Yellow solid (6 mg, 7%) with no distinct melting point. ^1^H NMR (DMSO-d_6_): 10.42 (1H, s), 9.98 (2H, s & br), 8.25 (1H, br), 8.03 (2H, d, *J* = 8.4 Hz), 7.97 (2H, d, *J* = 8.4 Hz), 7.92 (1H, d, *J* = 16.3 Hz), 7.76 (2H, t, *J* = 7.1 Hz), 7.50 (1H, d, *J* = 16.3 Hz), 7.42 (2H, t, *J* = 7.1 Hz), 7.35 (1H, d, *J* = 1.6 Hz), 7.22 (1H, d, *J* = 1.6 Hz), 7.13 (1H, d, *J* = 1.6 Hz), 6.99 (1H, d, *J* = 1.6 Hz), 4.02–3.99 (2H, m), 3.88 (3H, s), 3.83 (3H, s), 3.72–3.64 (2H, m), 3.55–3.54 (4H, m), 3.27 (2H, m), 3.14 (2H, m). IR (KBr): 3427, 1673, 1588, 1402, 1253, 1202, 1174, 832, 720 cm^−1^. HRFABMS: found: 622.2775 calculated for C_34_H_36_O_5_N_7_ 622.2772.

#### 4.3.25 *N*-[5-({[3-(dimethylamino)propyl]amino}carbonyl)-1-methyl-1*H*-pyrrol-3-yl]-4-{[(4-{[imino(3-isoquinolinyl)methyl]amino}-1-methyl-1*H*-pyrrol-2-yl)carbonyl]amino}-1-methyl-1*H*-pyrrole-2-carboxamide **17**

Yellow solid (26 mg, 41%) with no distinct melting point. ^1^H NMR (DMSO-d_6_): 10.02 (1H, s), 9.91 (1H, s), 9.60 (1H, s), 8.91 (1H, s), 8.40 (1H, d, *J* = 8.1 Hz), 8.20 (1H, d, *J* = 8.1 Hz), 8.03 (1H, t, *J* = 8.9 Hz), 7.98 (1H, t, *J* = 8.9 Hz), 7.32 (1H, d, *J* = 1.7 Hz), 7.26 (1H, d, *J* = 1.7 Hz), 7.16 (1H, d, *J* = 1.7 Hz), 7.11 (1H, d, *J* = 1.7 Hz), 7.08 (1H, d, *J* = 1.7 Hz), 6.95 (1H, d, *J* = 1.7 Hz), 3.97 (3H, s), 3.86 (3H, s), 3.81 (3H, s), 3.24 (2H, t, *J* = 6.4 Hz), 3.07 (2H, m), 2.79 (6H, s), 1.84 (2H, quintet, *J* = 7.6 Hz). IR (KBr): 1675, 1582, 1545, 1467, 1436, 1405, 1263, 1202, 1134, 802, 720 cm^−1^. HRFABMS: found: 623.3202 calculated for C_33_H_39_O_3_N_10_ 623.3207.

#### 4.3.26 *N*-[5-({[4-({[3-(Dimethylamino)propyl]amino}carbonyl)-5-isopentyl-1,3-thiazol-2-yl]amino}carbonyl)-1-methyl-1*H*-pyrrol-3-yl]-3-isoquinolinecarboxamide **18**

Prepared as per [19].

#### 4.3.27 *N*-[3-(Dimethylamino)propyl]-2-{[(4-{[(4-{[imino(3-isoquinolinyl)methyl]amino}-1-methyl-1*H*-pyrrol-2-yl)carbonyl]amino}-1-methyl-1*H*-pyrrol-2-yl)carbonyl]amino}-5-isopentyl-1,3-thiazole-4-carboxamide **19**

Prepared as per [19].

#### 4.3.28 5-isopentyl-2-({[1-methyl-4-({4-[(*E*)-2-(2-naphthyl)ethenyl]benzoyl}amino)-1*H*-pyrrol-2-yl]carbonyl}amino)-*N*-[2-(4-morpholinyl)ethyl]-1,3-thiazole-4-carboxamide **20**

Prepared as per [19].

#### 4.3.29 5-isopentyl-2-[({1-methyl-4-[({1-methyl-4-[(*E*)-2-(4-nitrophenyl)ethenyl]-1*H*-pyrrol-2-yl}carbonyl)amino]-1*H*-pyrrol-2-yl}carbonyl)amino]-*N*-[2-(4-morpholinyl)ethyl]-1,3-thiazole-4-carboxamide **21**

Prepared as per [22].

#### 4.3.30 1-methyl-*N*-[1-methyl-5-({[2-(4-morpholinyl)ethyl]amino}carbonyl)-1*H*-pyrrol-3-yl]-4-[(4-{(*E*)-2-[4-(trifluoromethyl)phenyl]ethenyl}benzoyl)amino]-1*H*-pyrrole-2-carboxamide **22**

Pale yellow solid (29.6 mg, 32%) with no distinct melting point. ^1^H NMR (DMSO-d_6_): 10.38 (1H, s), 10.01 (1H, s), 9.65 (1H, br), 8.26 (1H, t, *J* = 5.7 Hz), 8.01 (2H, d, *J* = 8.5 Hz), 7.88 (2H, d, *J* = 8.2 Hz), 7.81 (2H, d, *J* = 8.6 Hz), 7.78 (2H, d, *J* = 8.6 Hz), 7.52 (2H, s), 7.35 (1H, d, *J* = 1.8 Hz), 7.23 (1H, d, *J* = 1.8 Hz), 7.13 (1H, d, *J* = 1.8 Hz), 7.06 (1H, d, *J* = 1.8 Hz), 4.03 (2H, m), 3.88 (3H, s), 3.84 (3H, s), 3.69–3.15 (10H, m). IR (KBr): 1647, 1580, 1536, 1432, 1404, 1259, 1202, 1127 cm^−1^. HRFABMS: Found: 649.2747 calculated for C_34_H_36_O_4_N_6_F_3_ 649.2750.

#### 4.3.31 5-isopentyl-2-({[1-methyl-4-({4-[(*E*)-2-(2-quinolinyl)ethenyl]benzoyl}amino)-1*H*-pyrrol-2-yl]carbonyl}amino)-*N*-[2-(4-morpholinyl)ethyl]-1,3-thiazole-4-carboxamide **23**

Prepared as per [19].

#### 4.3.32 1-Methyl-*N*-[1-methyl-5-({[2-(4-morpholinyl)ethyl]amino}carbonyl)-1*H*-pyrrol-3-yl]-4-({4-[(*E*)-2-(4-pyridinyl)ethenyl]benzoyl}amino)-1*H*-pyrrole-2-carboxamide **24**

Prepared as per [19].

#### 4.3.33 4-[(2,4-Dichlorobenzoyl)amino]-1-methyl-*N*-[1-methyl-5-({[1-methyl-5-({[3-(4-morpholinyl)propyl]amino}carbonyl)-1*H*-pyrrol-3-yl]amino}carbonyl)-1*H*-pyrrol-3-yl]-1*H*-pyrrole-2-carboxamide **25**

Brown solid (45 mg, 51%) with no distinct melting point. ^1^H NMR (DMSO-d_6_): 10.48 (1H, s), 9.96 (1H, s), 9.89 (1H, s), 9.67 (1H, br), 8.15 (1H, t, *J* = 5.8 Hz), 7.74 (1H, d, *J* = 1.7 Hz), 7.54 (2H, m), 7.27 (1H, d, *J* = 1.7 Hz), 7.23 (1H, d, *J* = 1.7 Hz), 7.17 (1H, d, *J* = 1.7 Hz), 7.06 (1H, d, *J* = 1.7 Hz), 7.02 (1H, d, *J* = 1.7 Hz), 6.95 (1H, d, *J* = 1.7 Hz), 3.97–3.89 (2H, m), 3.90 (3H, s), 3.84 (3H, s), 3.81 (3H, s), 3.71–3.64 (2H, m), 3.61–3.26 (2H, m), 3.26 (2H, q, *J* = 6.0 Hz), 3.12–3.06 (4H, m), 1.90–1.85 (2H, quintet, *J* = 7.1 Hz). IR (KBr): 1665, 1550, 1290, 1200, 1131 cm^−1^. HRFABMS: found: 683.2275 calculated for C_32_H_37_^35^Cl_2_N_8_O_5_ 683.2264.

#### 4.3.34 4-({4-[(*E*)-2-(4-fluorophenyl)ethenyl]benzoyl}amino)-1-methyl-*N*-[1-methyl-5-({[2-(4-morpholinyl)ethyl]amino}carbonyl)-1*H*-pyrrol-3-yl]-1*H*-pyrrole-2-carboxamide **26**

Yellow solid after freeze drying (54 mg, 63%) with no distinct melting point. ^1^H NMR (DMSO-d_6_): 10.32 (1H, s), 9.98 (1H, s), 9.54 (1H, br), 8.23 (1H, t, *J* = 5.6 Hz), 7.97 (2H, d, *J* = 8.4 Hz), 7.74 (2H, d, *J* = 8.4 Hz), 7.71–7.68 (2H, m), 7.43 (1H, d, *J* = 23.2 Hz), 7.39–7.21 (5H, m), 7.12 (1H, d, *J* = 1.8 Hz), 7.01 (1H, d, *J* = 1.8 Hz), 4.03 (2H, m), 3.88 (3H, s), 3.84 (3H, s), 3.69–3.15 (10H, m). IR (KBr): 1647, 1580, 1536, 1432, 1404, 1259, 1202, 1127 cm^−1^. HRFABMS: Found: 599.2785 calculated for C_33_H_36_O_4_N_6_F 599.2782.

#### 4.3.35 4-({4-[(*E*)-2-(3-fluorophenyl)ethenyl]benzoyl}amino)-1-methyl-*N*-[1-methyl-5-({[2-(4-morpholinyl)ethyl]amino}carbonyl)-1*H*-pyrrol-3-yl]-1*H*-pyrrole-2-carboxamide **27**

Pale yellow solid (41 mg, 45%) with no distinct melting point. ^1^H NMR (DMSO-d_6_): 10.34 (1H, s), 9.98 (1H, s), 9.58 (1H, br), 8.24 (1H, t, *J* = 5.7 Hz), 7.99 (2H, d, *J* = 8.5 Hz), 7.76 (2H, d, *J* = 8.5 Hz), 7.54–7.44 (3H, m), 7.42 (2H, s), 7.34 (1H, d, *J* = 1.8 Hz), 7.22 (1H, d, *J* = 1.8 Hz), 7.17–7.11 (2H, m), 7.01 (1H, d, *J* = 1.8 Hz), 4.03 (2H, m), 3.88 (3H, s), 3.84 (3H, s), 3.69–3.15 (10H, m). IR (KBr): 1647, 1580, 1536, 1432, 1404, 1259, 1202, 1127 cm^−1^. HRFABMS: Found: 599.2790 calculated for C_33_H_36_O_4_N_6_F 599.2782.

#### 4.3.36 4-({4-[(*E*)-2-(2,1,3-benzothiadiazol-5-yl)ethenyl]benzoyl}amino)-1-methyl-*N*-[1-methyl-5-({[2-(4-morpholinyl)ethyl]amino}carbonyl)-1*H*-pyrrol-3-yl]-1*H*-pyrrole-2-carboxamide **28**

Yellow solid (12 mg, 13%) with no distinct melting point. ^1^H NMR (DMSO-d_6_): 10.39 (1H, s), 10.01 (1H, s), 9.55 (1H, br), 10.45–10.38 (3H, m), 10.34 (1H, d, *J* = 9.0 Hz), 10.22–10.20 (2H, d, *J* = 8.4 Hz), 10.04 (2H, d, *J* = 8.4 Hz), 9.87 (2H, s), 9.55 (1H, d, *J* = 1.7 Hz), 9.42 (1H, d, *J* = 1.7 Hz), 9.34 (1H, d, *J* = 1.7 Hz), 9.21 (1H, br), 4.03 (2H, m), 3.88 (3H, s), 3.84 (3H, s), 3.69–3.15 (10H, m). IR (KBr): 1647, 1580, 1536, 1432, 1404, 1259, 1202, 1127 cm^−1^. HRFABMS: Found: 639.2498 calculated for C_33_H_35_O_4_N_8_S 639.2502.

#### 4.3.37 4-({4-[(*E*)-2-(2,1,3-benzoxadiazol-5-yl)ethenyl]benzoyl}amino)-1-methyl-*N*-[1-methyl-5-({[2-(4-morpholinyl)ethyl]amino}carbonyl)-1*H*-pyrrol-3-yl]-1*H*-pyrrole-2-carboxamide **29**

Yellow solid (30 mg, 30%) with no distinct melting point. ^1^H NMR (DMSO-d_6_): 10.40 (1H. s), 10.01 (1H, s), 9.53 (1H, br), 8.25 (1H, t, *J* = 5.9 Hz), 8.11 (3H, s), 8.03 (2H, d, *J* = 8.4 Hz), 7.83 (2H, d, *J* = 8.4 Hz), 7.73 (1H, d, *J* = 16.4 Hz), 7.64 (1H, d, *J* = 16.4 Hz), 7.35 (1H, d, *J* = 1.7 Hz), 7.22 (1H, d, *J* = 1.7 Hz), 7.14 (1H, d, *J* = 1.7 Hz), 7.02 (1H, d, *J* = 1.7 Hz), 4.03 (2H, m), 3.88 (3H, s), 3.84 (3H, s), 3.69–3.15 (10H, m). IR (KBr): 1647, 1580, 1536, 1432, 1404, 1259, 1202, 1127 cm^−1^. HRFABMS: Found: 623.2728 calculated for C_33_H_35_O_5_N_8_ 623.2730.

#### 4.3.38 2-[(*E*)-2-(4-Methoxyphenyl)ethenyl]-*N*-[1-methyl-5-({[1-methyl-5-({[2-(4-morpholinyl)ethyl]amino}carbonyl)-1*H*-pyrrol-3-yl]amino}carbonyl)-1*H*-pyrrol-3-yl]-6-quinolinecarboxamide **30**

Prepared as per [19].

#### 4.3.39 *N*-[1-methyl-5-({[1-methyl-5-({[2-(4-morpholinyl)ethyl]amino}carbonyl)-1*H*-pyrrol-3-yl]amino}carbonyl)-1*H*-pyrrol-3-yl]-2-{(*E*)-2-[4-(methylsulfanyl)phenyl]ethenyl}-6-quinolinecarboxamide **31**

Prepared as per [19].

#### 4.3.40 6-{(*E*)-2-[4-(Dimethylamino)phenyl]ethenyl}-*N*-[1-methyl-5-({[1-methyl-5-({[2-(4-morpholinyl)ethyl]amino}carbonyl)-1*H*-pyrrol-3-yl]amino}carbonyl)-1*H*-pyrrol-3-yl]nicotinamide **32**

Red solid (69 mg, 38%) with no distinct melting point. ^1^H NMR (DMSO-d_6_): 10.44 (1H, s), 9.99 (1H, s), 9.54 (1H, br), 9.05 (1H, d, *J* = 2.0 Hz), 8.26–8.22 (2H, m), 7.74 (1H, d, *J* = 16.0 Hz), 7.64 (1H, d, *J* = 8.4 Hz), 7.55 (1H, s), 7.53 (1H, s), 7.35 (1H, d, *J* = 1.7 Hz), 7.22 (1H, d, *J* = 1.7 Hz), 7.14–7.09 (2H, m), 7.02 (1H, d, *J* = 1.7 Hz), 6.78 (1H, s), 6.76 (1H, s), 4.04–4.01 (2H, m), 3.89 (3H, s), 3.85 (3H, s), 3.70–3.55 (6H, m), 3.30–3.29 (2H, m), 3.16–3.13 (2H, m), 2.98 (6H, s). IR (KBr): 721, 804, 1130, 1173, 1289, 1368, 1435, 1530, 1576, 1665 cm^−1^. HRFABMS: Found: 623.3209 calculated for C_33_H_39_O_3_N_10_ 623.3201.

## Figures and Tables

**Table 1:** Activities against *Trypanosoma brucei brucei*. Where N/A is an IC_50_ > 75 μM and all standard deviations are less than 2%. Diminazene positive control has an IC_50_ of 5 nM *Compounds determined to have significant activity.

Compound	IC_50_ (μM)	Compound	IC_50_ (μM)
**1**	N/A	**17**	4.9
**2**	N/A	**18**	0.39
**3**	0.40	**19**	2.2
**4**	48	**20**	0.040*
**5**	N/A	**21**	0.0054*
**6**	4.0	**22**	0.73
**7**	27	**23**	0.62
**8**	0.0068*	**24**	0.38
**9**	N/A	**25**	2.3
**10**	0.30	**26**	0.71
**11**	0.28	**27**	0.22
**12**	0.18	**28**	0.0073*
**13**	0.21	**29**	0.019*
**14**	0.97	**30**	0.63
**15**	2.2	**31**	5.0
**16**	0.79	**32**	0.17

**Table 2:** Activities against HEK 293 cells and *Trypanosoma brucei brucei*. Where N/A is an IC_50_ value > 20 μM. The Puromycin control had an IC_50_of 350.5 nM (±2.12) from two separate independent experiments. *Calculation of selectivity index (SI) was made using 20 μM as the IC_50_ value for HEK 293 cells in all cases apart from compound 21 where an estimated IC_50_ of 10 μM was made based on an average of 54% inhibition at 20 μM.

Compound	% HEK 293 Inhibition at 20 μM	*Trypanosoma brucei brucei* IC_50_ (μM)	SI*
**8**	N/A	0.0068	>2941
**20**	N/A	0.040	>500
**21**	54	0.0054	>1851*
**28**	N/A	0.0073	>2739
**29**	N/A	0.019	>1052
