# Supplementary material for: An evaluation of Minor Groove Binders as anti-Trypanosoma brucei brucei therapeutics
Source: Eur J Med Chem. 2016 Jun 30;116:116–25. doi: 10.1016/j.ejmech.2016.03.064 (PMC4872591; doi:10.1016/j.ejmech.2016.03.064)
Supplement: Supplementary file 1 [file mmc1.docx]

***Electronic supplementary information (ESI)***

An Evaluation of Minor Groove Binders as anti-*Trypanosma brucei brucei* Therapeutics

Fraser J. Scott^a^, Abedawn I. Khalaf^a^, Federica Giordani^b^, Pui Ee Wong^b^, Mike Barrett^b^ and Colin J. Suckling^a^

^a^WestCHEM Department of Pure and Applied Chemistry, University of Strathclyde, 295 Cathedral Street, Glasgow G1 1XL, United Kingdom

^b^Wellcome Trust Centre for Molecular Parasitology, Institute of Infection, Immunity and Inflammation and Glasgow Polyomics, College of Medical, Veterinary and Life Sciences, University of Glasgow, Glasgow G12 8TA, United Kingdom

**Contents**

# 1. Minor Groove Binder Structures

Table S1. MGB Structrues

*Page 3*

# 1. Lipohilicity Measurements

Table S2. LogD values at various pHs

*Page 5*

Figure S1. LogD_5.0_ against MGB Activity.

*Page 6*

Figure S2. LogD_6.5_ against MGB Activity.

*Page 6*

Figure S3. LogD1.5 against MGB Activity.

*Page 7*

# 2. Exemplar NMR Spectra

^1^H NMR spectrum of *N*-[3-(Dimethylamino)propyl]-5-isopentyl-2-({[4-({3-[(*E*)-2-(3-methoxyphenyl)ethenyl]benzoyl}amino)-1-methyl-1*H*-pyrrol-2-yl]carbonyl}amino)-1,3-thiazole-4-carboxamide **12**

*Page 8*

^1^H NMR spectrum of *N*-[3-(dimethylamino)propyl]-5-isopentyl-2-[({1-methyl-4-[({1-methyl-4-[(*E*)-2-(4-nitrophenyl)ethenyl]-1*H*-pyrrol-2-yl}carbonyl)amino]-1*H*-pyrrol-2-yl}carbonyl)amino]-1,3-thiazole-4-carboxamide **13**

*Page 9*

^1^H NMR spectrum of 4-({4-[(*E*)-2-(1,3-benzoxazol-2-yl)ethenyl]benzoyl}amino)-1-methyl-*N*-[1-methyl-5-({[2-(4-morpholinyl)ethyl]amino}carbonyl)-1*H*-pyrrol-3-yl]-1*H*-pyrrole-2-carboxamide **16**

*Page 10*

^1^H NMR spectrum of *N*-[5-({[3-(dimethylamino)propyl]amino}carbonyl)-1-methyl-1*H*-pyrrol-3-yl]-4-{[(4-{[imino(3-isoquinolinyl)methyl]amino}-1-methyl-1*H*-pyrrol-2-yl)carbonyl]amino}-1-methyl-1*H*-pyrrole-2-carboxamide **17**

*Page 11*

^1^H NMR spectrum of *N*-[3-(Dimethylamino)propyl]-2-{[(4-{[(4-{[imino(3-isoquinolinyl)methyl]amino}-1-methyl-1*H*-pyrrol-2-yl)carbonyl]amino}-1-methyl-1*H*-pyrrol-2-yl)carbonyl]amino}-5-isopentyl-1,3-thiazole-4-carboxamide **19**

*Page 12*

^1^H NMR spectrum of 5-isopentyl-2-({[1-methyl-4-({4-[(*E*)-2-(2-naphthyl)ethenyl]benzoyl}amino)-1*H*-pyrrol-2-yl]carbonyl}amino)-*N*-[2-(4-morpholinyl)ethyl]-1,3-thiazole-4-carboxamide **20**

*Page 13*

^1^H NMR spectrum of 5-isopentyl-2-[({1-methyl-4-[({1-methyl-4-[(*E*)-2-(4-nitrophenyl)ethenyl]-1*H*-pyrrol-2-yl}carbonyl)amino]-1*H*-pyrrol-2-yl}carbonyl)amino]-*N*-[2-(4-morpholinyl)ethyl]-1,3-thiazole-4-carboxamide **21**

*Page 14*

^1^H NMR spectrum of 1-methyl-*N*-[1-methyl-5-({[2-(4-morpholinyl)ethyl]amino}carbonyl)-1*H*-pyrrol-3-yl]-4-[(4-{(*E*)-2-[4-(trifluoromethyl)phenyl]ethenyl}benzoyl)amino]-1*H*-pyrrole-2-carboxamide **22**

*Page 15*

^1^H NMR spectrum of 5-isopentyl-2-({[1-methyl-4-({4-[(*E*)-2-(2-quinolinyl)ethenyl]benzoyl}amino)-1*H*-pyrrol-2-yl]carbonyl}amino)-*N*-[2-(4-morpholinyl)ethyl]-1,3-thiazole-4-carboxamide **23**

*Page 16*

^1^H NMR spectrum of 4-({4-[(*E*)-2-(4-fluorophenyl)ethenyl]benzoyl}amino)-1-methyl-*N*-[1-methyl-5-({[2-(4-morpholinyl)ethyl]amino}carbonyl)-1*H*-pyrrol-3-yl]-1*H*-pyrrole-2-carboxamide **26**

*Page 17*

^1^H NMR spectrum of 4-({4-[(*E*)-2-(3-fluorophenyl)ethenyl]benzoyl}amino)-1-methyl-*N*-[1-methyl-5-({[2-(4-morpholinyl)ethyl]amino}carbonyl)-1*H*-pyrrol-3-yl]-1*H*-pyrrole-2-carboxamide **27**

*Page 18*

^1^H NMR spectrum of 2-[(*E*)-2-(4-Methoxyphenyl)ethenyl]-*N*-[1-methyl-5-({[1-methyl-5-({[2-(4-morpholinyl)ethyl]amino}carbonyl)-1*H*-pyrrol-3-yl]amino}carbonyl)-1*H*-pyrrol-3-yl]-6-quinolinecarboxamide **30**

*Page 19*

# 3. Novel MGB Fragments

Diethyl 2,1,3-benzothiadiazol-5-ylmethylphosphonate

*Page 20*

Methyl 4-[(*E*)-2-(2,1,3-benzothiadiazol-5-yl)ethenyl]benzoate

*Page 20*

4-[(*E*)-2-(2,1,3-benzothiadiazol-5-yl)ethenyl]benzoic acid

*Page 20*

Diethyl 2,1,3-benzoxadiazol-5-ylmethylphosphonate

*Page 21*

Methyl 4-[(*E*)-2-(2,1,3-benzoxadiazol-5-yl)ethenyl]benzoate

*Page 21*

4-[(*E*)-2-(2,1,3-Benzoxadiazol-5-yl)ethenyl]benzoic acid

*Page 22*

4-{(*E*)-2-[4-(dimethylamino)phenyl]ethenyl}benzoic acid

*Page 22*

# 4. References

Van der Eycken *et al.* (2002)

*Page 22*

# 1. Minor Groove Binder Structures

| **Compound** | **Structure** | **Compound** | **Structure** |
| --- | --- | --- | --- |
| **1** |  | **17** |  |
| **2** |  | **18** |  |
| **3** |  | **19** |  |
| **4** |  | **20** |  |
| **5** |  | **21** |  |
| **6** |  | **22** |  |
| **7** |  | **23** |  |
| **8** |  | **24** |  |
| **9** |  | **25** |  |
| **10** |  | **26** |  |
| **11** |  | **27** |  |
| **12** |  | **28** |  |
| **13** |  | **29** |  |
| **14** |  | **30** |  |
| **15** |  | **31** |  |
| **16** |  | **32** |  |

# 2. Lipophilicity Measurements

Table S2 shows the logD values for each compound at all four pHs investigated. Figures S1 to S3 show these logD values plotted against their activites; that for pH 7.4 is not shown as it is in the main manuscript.

| **Compound** | **IC_50_ (µM)** | logD_1.5_ | logD_5.0_ | logD_6.5_ | logD_7.4_ |
| --- | --- | --- | --- | --- | --- |
| **1** | N/A | -3.83 | -3.62 | -1.93 | -0.35 |
| **2** | N/A | -5.69 | -5.61 | -4.68 | -3.28 |
| **3** | 0.40 | -0.46 | 1.97 | 2.89 | 2.99 |
| **4** | 48 | -3.57 | -1.14 | -0.22 | -0.13 |
| **5** | N/A | -2.31 | 0.58 | 2.48 | 3.2 |
| **6** | 4.0 | -0.45 | -0.38 | 0.34 | 1.16 |
| **7** | 27 | -3.22 | -2.87 | -1 | 0.48 |
| **8** | 0.0068 | -1.53 | 0 | 1.37 | 1.86 |
| **9** | N/A | -4.2 | -2.44 | 0.09 | 1.23 |
| **10** | 2.3 | -0.44 | 1.05 | 2.42 | 2.91 |
| **11** | 0.30 | 2.2 | 3.53 | 4.3 | 5.13 |
| **12** | 0.28 | 3.21 | 3.27 | 3.99 | 4.82 |
| **13** | 0.18 | 3.21 | 3.27 | 3.99 | 4.82 |
| **14** | 0.21 | 2.53 | 2.6 | 3.31 | 4.14 |
| **15** | 0.97 | -1.87 | 0.61 | 1.52 | 1.62 |
| **16** | 2.2 | -1.64 | -1.52 | -0.81 | 0.02 |
| **17** | 4.9 | -3.64 | -1.57 | -0.5 | 0.35 |
| **18** | 0.39 | 1.14 | 1.25 | 1.97 | 2.79 |
| **19** | 2.2 | -0.87 | 1.2 | 2.28 | 3.12 |
| **20** | 0.040 | 4.15 | 6.76 | 7.51 | 7.57 |
| **21** | 0.0054 | 2.32 | 4.93 | 5.68 | 5.74 |
| **22** | 0.73 | 1.24 | 3.67 | 4.58 | 4.68 |
| **23** | 0.62 | 1.82 | 6.12 | 6.91 | 6.97 |
| **24** | 0.38 | -1.84 | 1.18 | 2.46 | 2.58 |
| **25** | 0.79 | -0.09 | 2.36 | 3.27 | 3.37 |
| **26** | 0.71 | 0.5 | 2.93 | 3.85 | 3.94 |
| **27** | 0.22 | 0.5 | 2.93 | 3.85 | 3.94 |
| **28** | 0.0073 | 0.5 | 2.93 | 3.85 | 3.95 |
| **29** | 0.019 | -0.29 | 2.14 | 3.05 | 3.15 |
| **30** | 0.63 | -1.04 | 3.01 | 3.94 | 4.04 |
| **31** | 5.0 | -0.26 | 3.79 | 4.72 | 4.82 |
| **32** | 0.17 | -0.69 | 1.74 | 2.65 | 2.74 |

**Table S1. LogD values at various pHs**

**Figure S1. LogD_5.0_ against MGB Activity.**

**Figure S2. LogD_6.5_ against MGB Activity.**

**Figure S3. LogD_1.5_ against MGB Activity.**

# 2. Exemplar NMR Spectra

^1^H NMR spectrum of *N*-[3-(Dimethylamino)propyl]-5-isopentyl-2-({[4-({3-[(*E*)-2-(3-methoxyphenyl)ethenyl]benzoyl}amino)-1-methyl-1*H*-pyrrol-2-yl]carbonyl}amino)-1,3 thiazole-4-carboxamide **12**
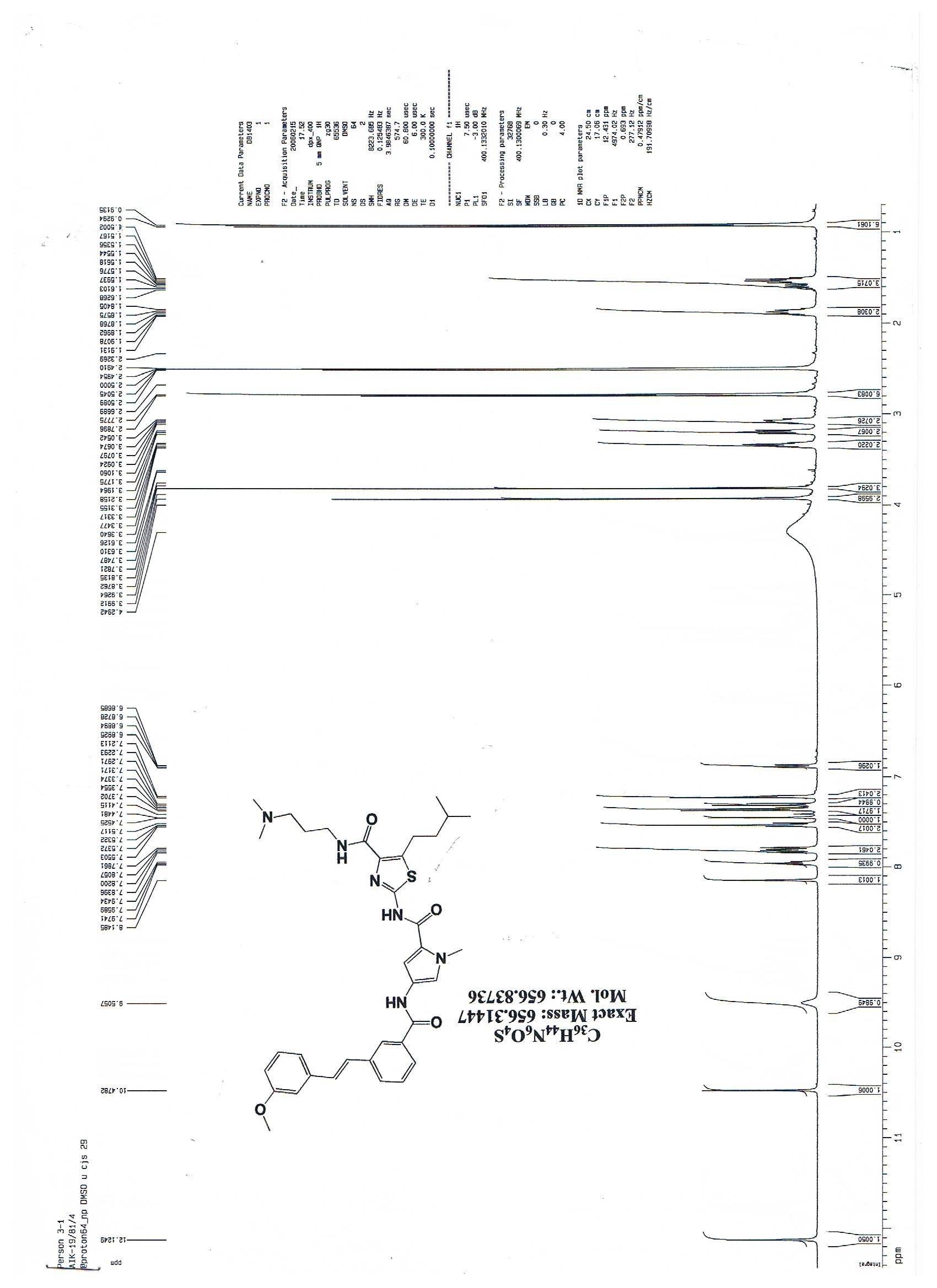


^1^H NMR spectrum of *N*-[3-(dimethylamino)propyl]-5-isopentyl-2-[({1-methyl-4-[({1-methyl-4-[(*E*)-2-(4-nitrophenyl)ethenyl]-1*H*-pyrrol-2-yl}carbonyl)amino]-1*H*-pyrrol-2-yl}carbonyl)amino]-1,3-thiazole-4-carboxamide **13**
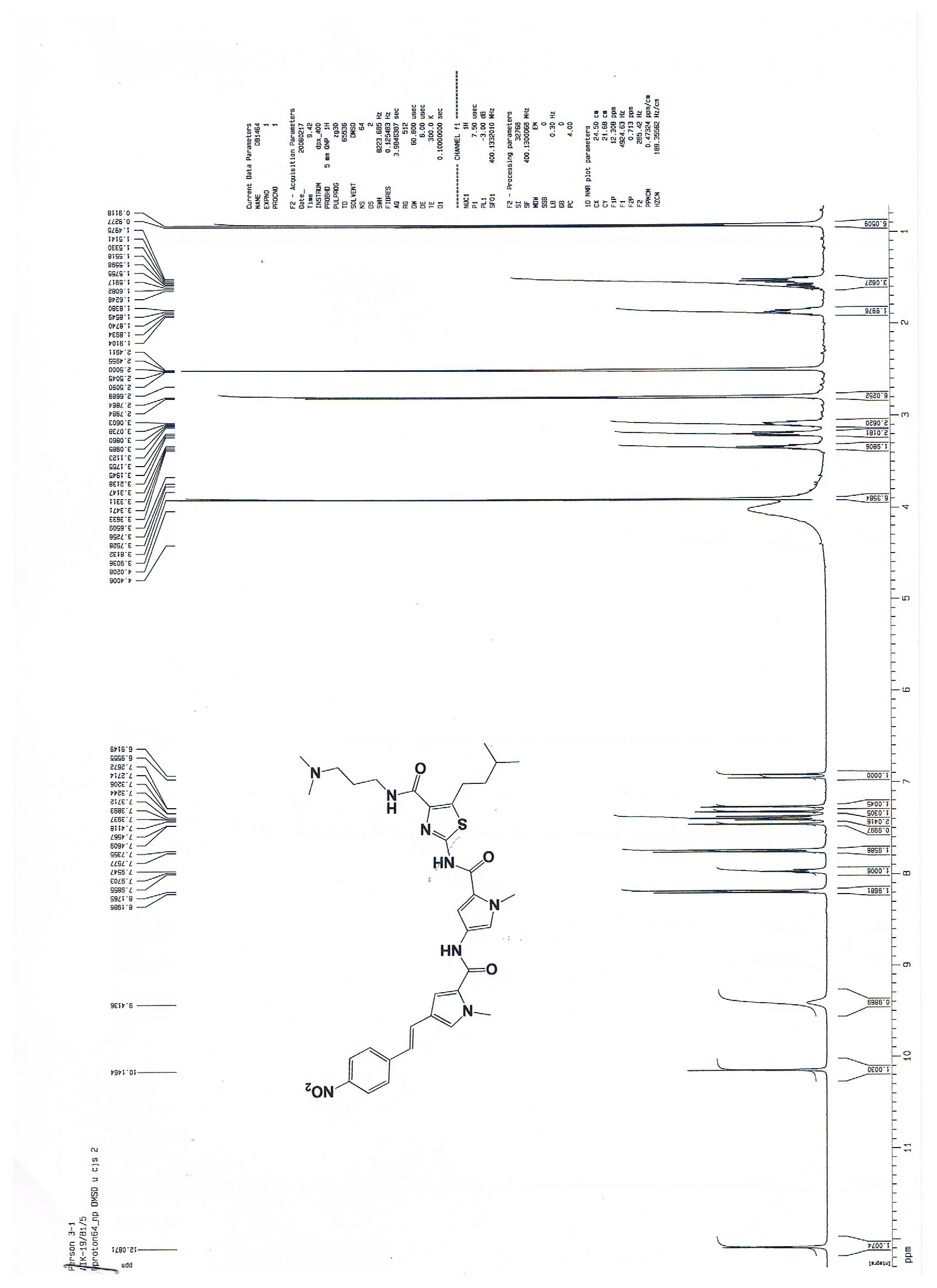


^1^H NMR spectrum of 4-({4-[(*E*)-2-(1,3-benzoxazol-2-yl)ethenyl]benzoyl}amino)-1-methyl-*N*-[1-methyl-5-({[2-(4-morpholinyl)ethyl]amino}carbonyl)-1*H*-pyrrol-3-yl]-1*H*-pyrrole-2-carboxamide **16**


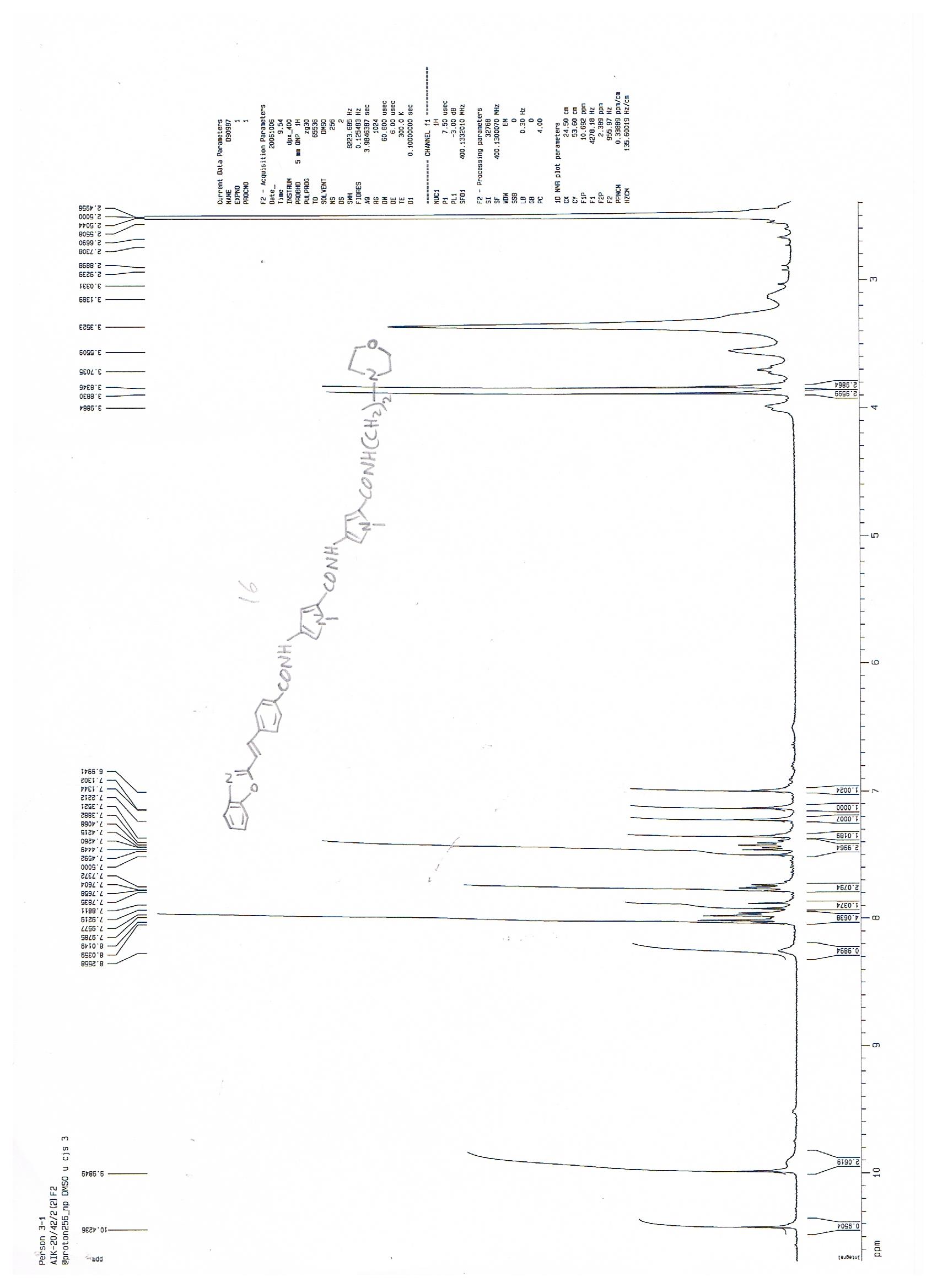
^1^H NMR spectrum of *N*-[5-({[3-(dimethylamino)propyl]amino}carbonyl)-1-methyl-1*H*-pyrrol-3-yl]-4-{[(4-{[imino(3-isoquinolinyl)methyl]amino}-1-methyl-1*H*-pyrrol-2-yl)carbonyl]amino}-1-methyl-1*H*-pyrrole-2-carboxamide **17**


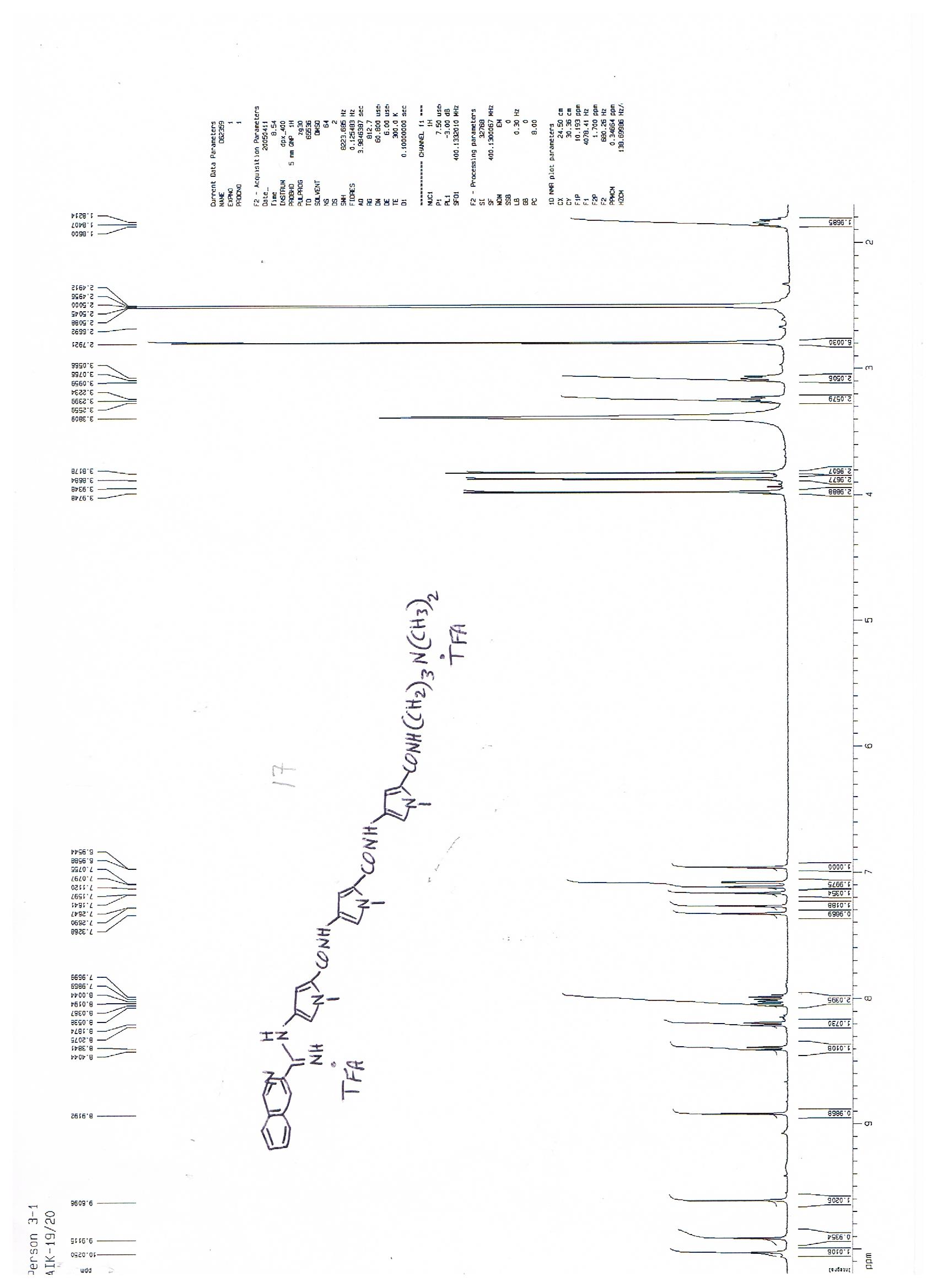
^1^H NMR spectrum of *N*-[3-(Dimethylamino)propyl]-2-{[(4-{[(4-{[imino(3-isoquinolinyl)methyl]amino}-1-methyl-1*H*-pyrrol-2-yl)carbonyl]amino}-1-methyl-1*H*-pyrrol-2-yl)carbonyl]amino}-5-isopentyl-1,3-thiazole-4-carboxamide **19**


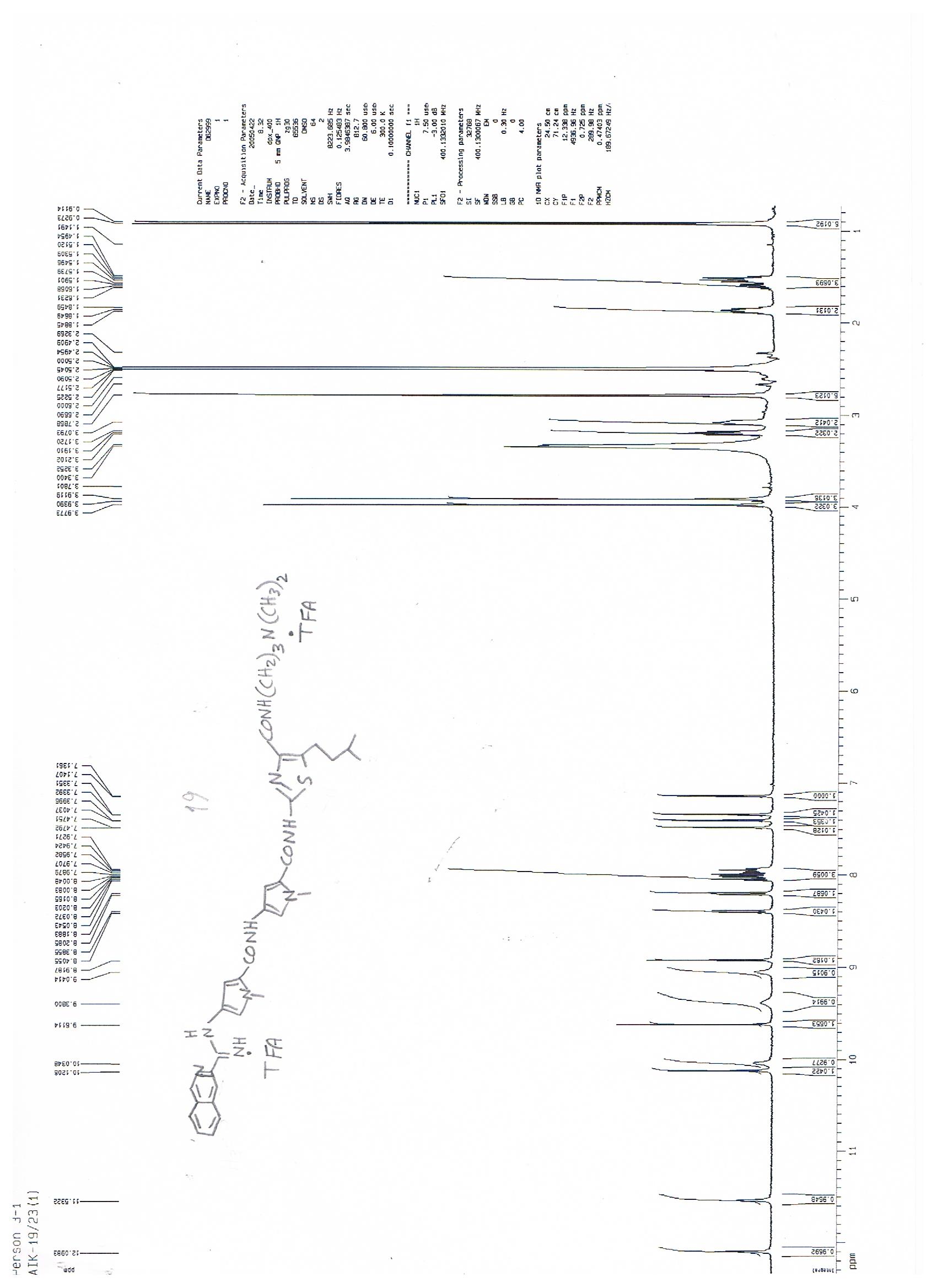
^1^H NMR spectrum of 5-isopentyl-2-({[1-methyl-4-({4-[(*E*)-2-(2-naphthyl)ethenyl]benzoyl}amino)-1*H*-pyrrol-2-yl]carbonyl}amino)-*N*-[2-(4-morpholinyl)ethyl]-1,3-thiazole-4-carboxamide **20**


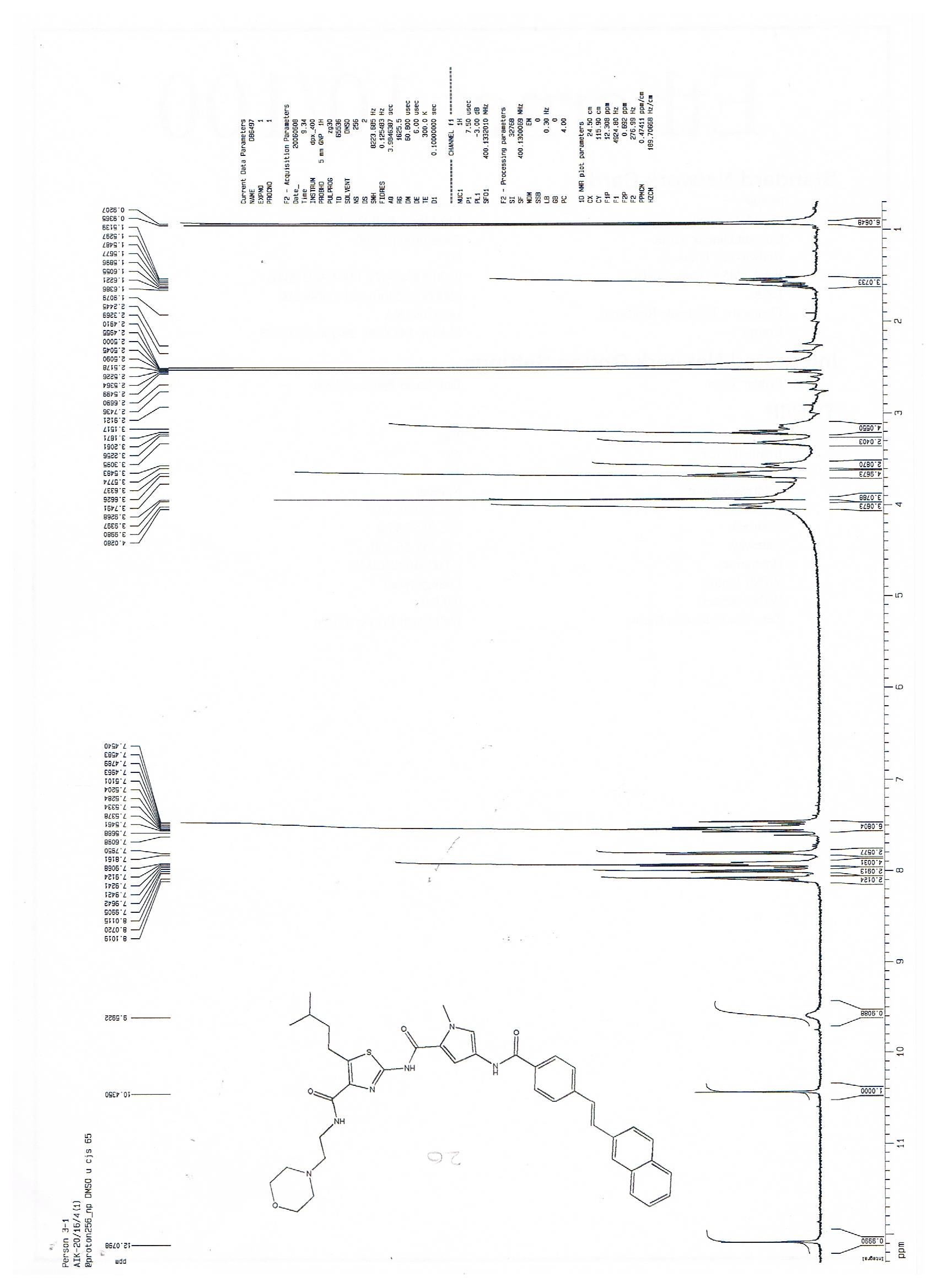
^1^H NMR spectrum of 5-isopentyl-2-[({1-methyl-4-[({1-methyl-4-[(*E*)-2-(4-nitrophenyl)ethenyl]-1*H*-pyrrol-2-yl}carbonyl)amino]-1*H*-pyrrol-2-yl}carbonyl)amino]-*N*-[2-(4-morpholinyl)ethyl]-1,3-thiazole-4-carboxamide **21**


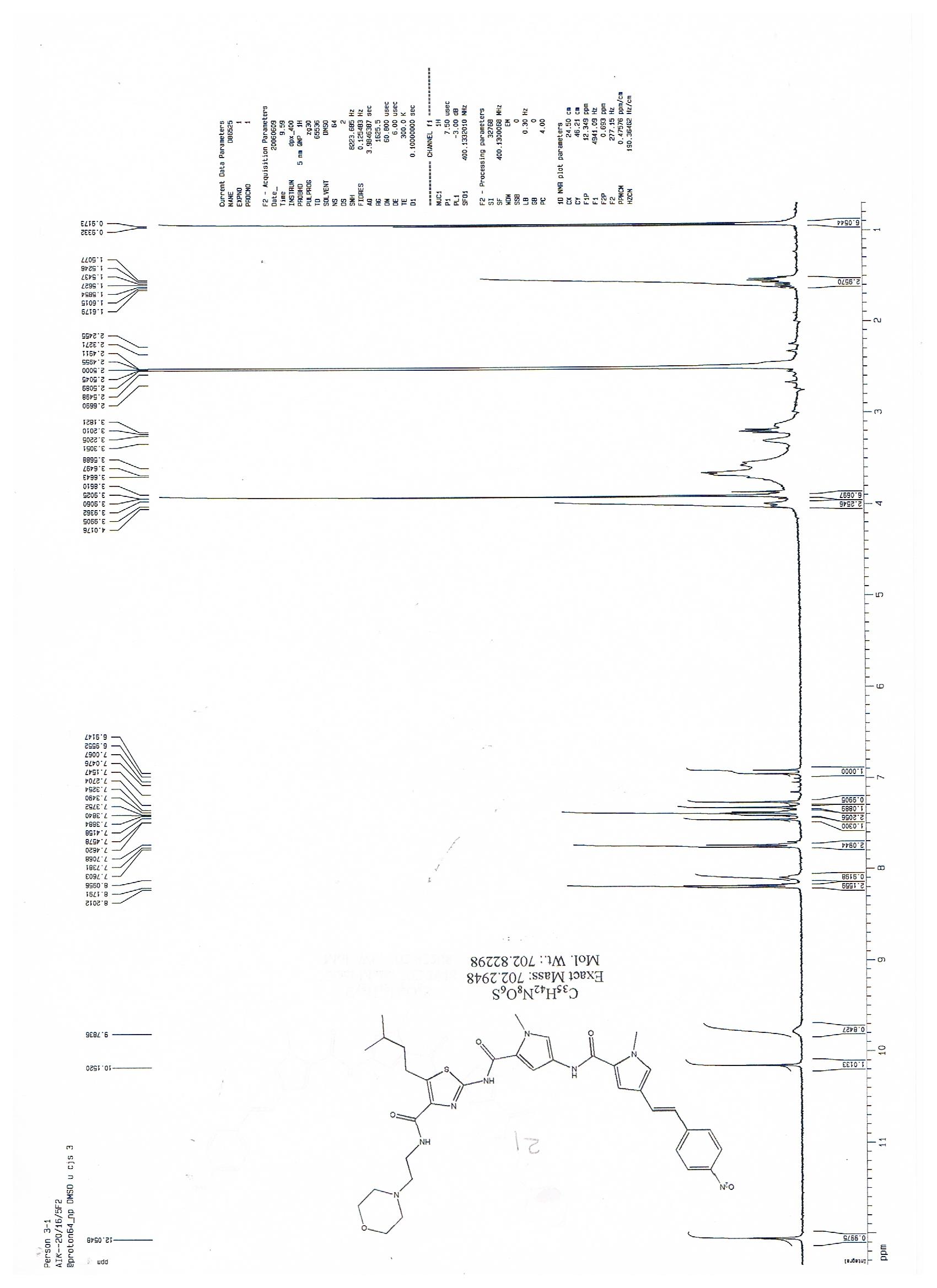
^1^H NMR spectrum of 1-methyl-*N*-[1-methyl-5-({[2-(4-morpholinyl)ethyl]amino}carbonyl)-1H-pyrrol-3-yl]-4-[(4-{(*E*)-2-[4-(trifluoromethyl)phenyl]ethenyl}benzoyl)amino]-1*H*-pyrrole-2-carboxamide **22**


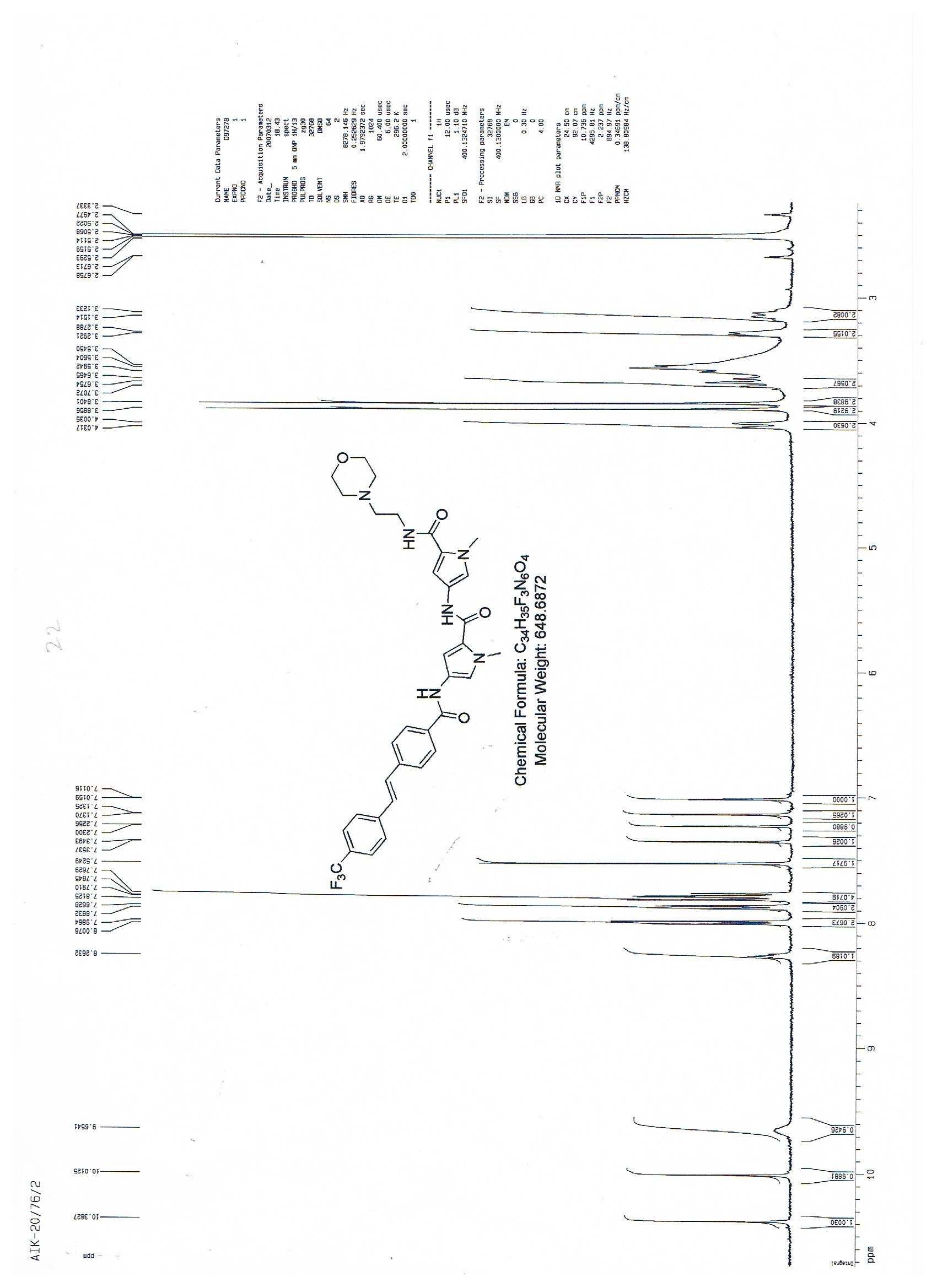
^1^H NMR spectrum of 5-isopentyl-2-({[1-methyl-4-({4-[(*E*)-2-(2-quinolinyl)ethenyl]benzoyl}amino)-1*H*-pyrrol-2-yl]carbonyl}amino)-*N*-[2-(4-morpholinyl)ethyl]-1,3-thiazole-4-carboxamide **23**


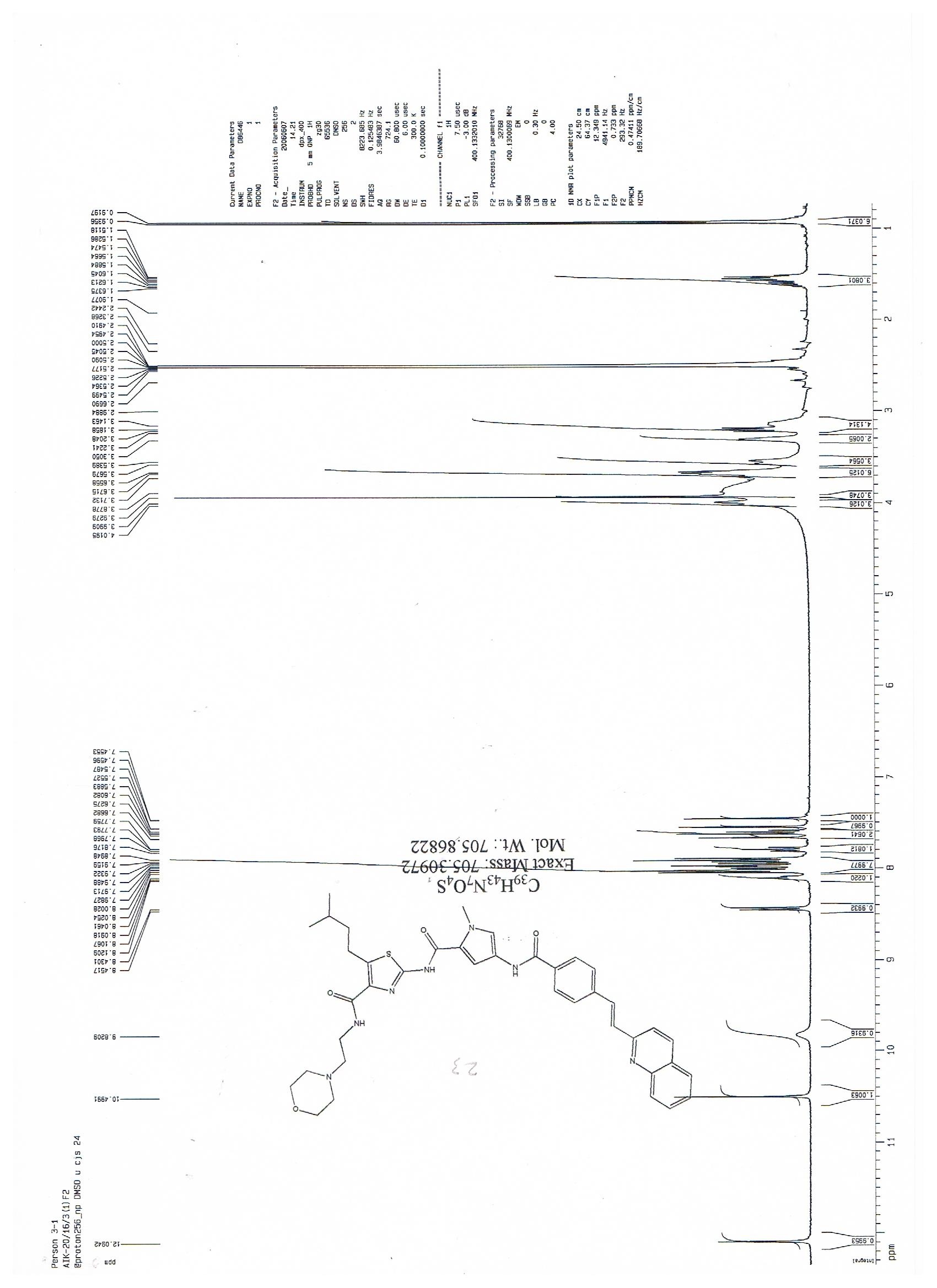
^1^H NMR spectrum of 4-({4-[(*E*)-2-(4-fluorophenyl)ethenyl]benzoyl}amino)-1-methyl-*N*-[1-methyl-5-({[2-(4-morpholinyl)ethyl]amino}carbonyl)-1*H*-pyrrol-3-yl]-1*H*-pyrrole-2-carboxamide **26**


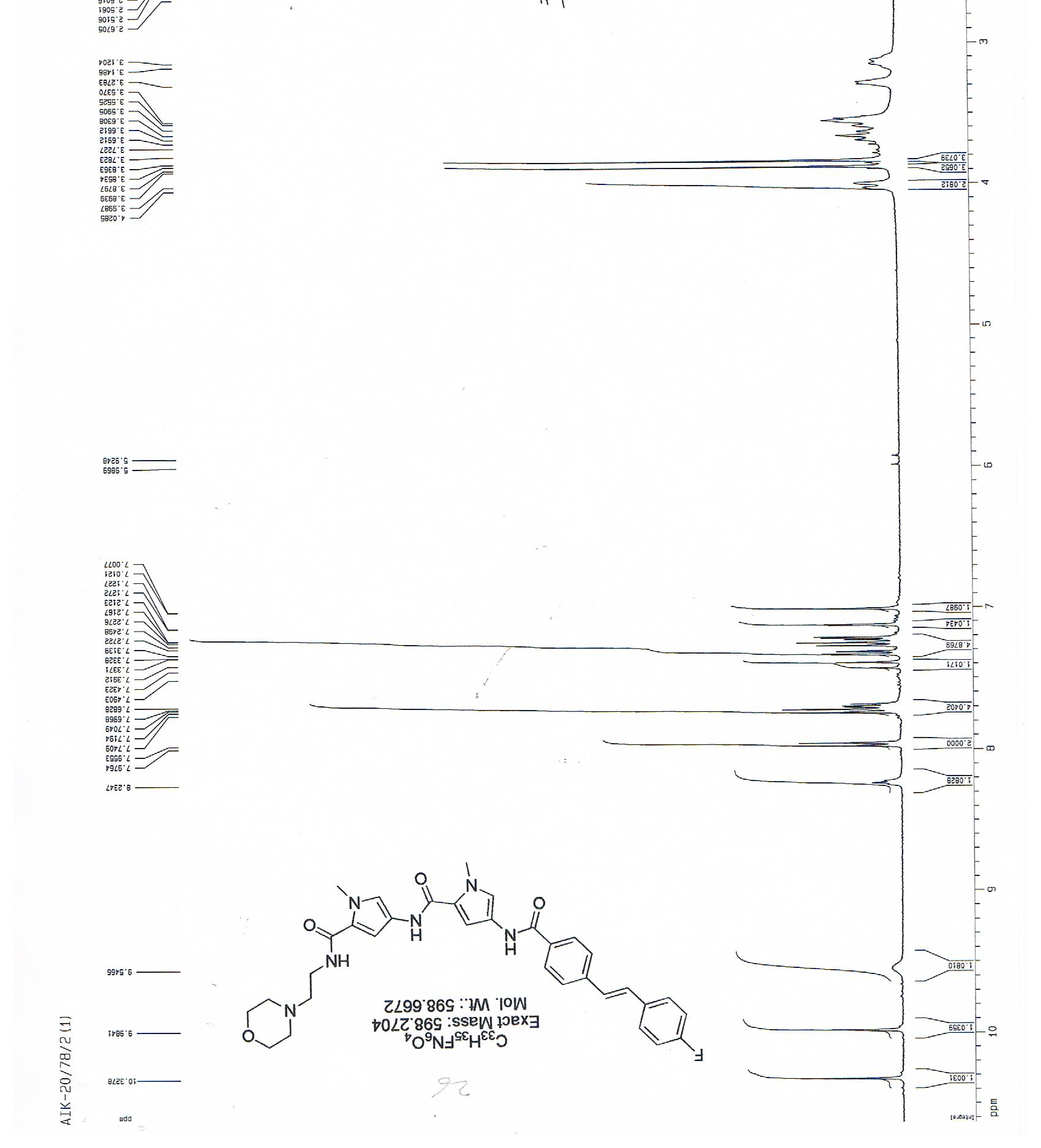


^1^H NMR spectrum of 4-({4-[(*E*)-2-(3-fluorophenyl)ethenyl]benzoyl}amino)-1-methyl-*N*-[1-methyl-5-({[2-(4-morpholinyl)ethyl]amino}carbonyl)-1*H*-pyrrol-3-yl]-1*H*-pyrrole-2-carboxamide **27**


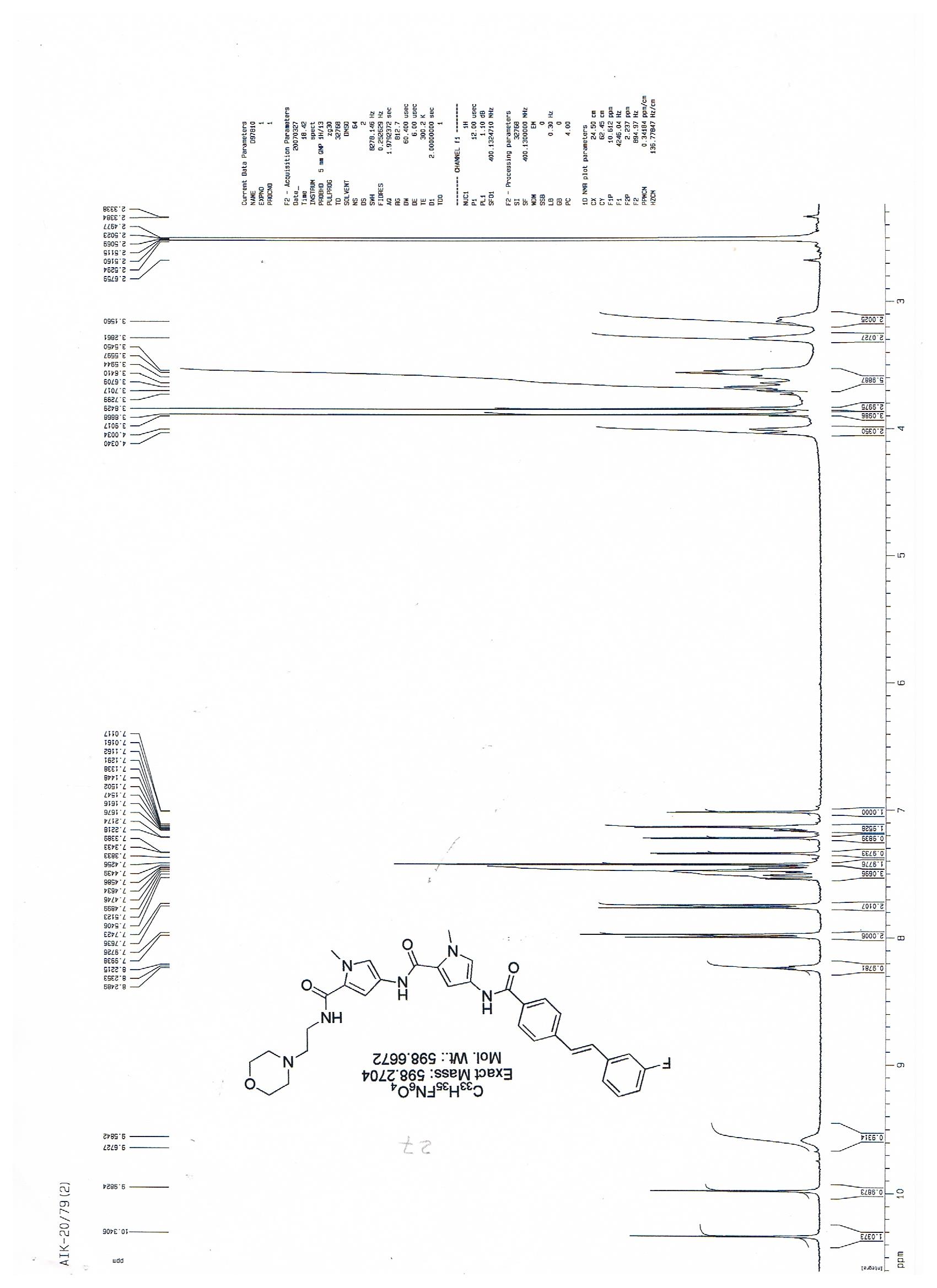
^1^H NMR spectrum of 2-[(*E*)-2-(4-Methoxyphenyl)ethenyl]-*N*-[1-methyl-5-({[1-methyl-5-({[2-(4-morpholinyl)ethyl]amino}carbonyl)-1*H*-pyrrol-3-yl]amino}carbonyl)-1*H*-pyrrol-3-yl]-6-quinolinecarboxamide **30**


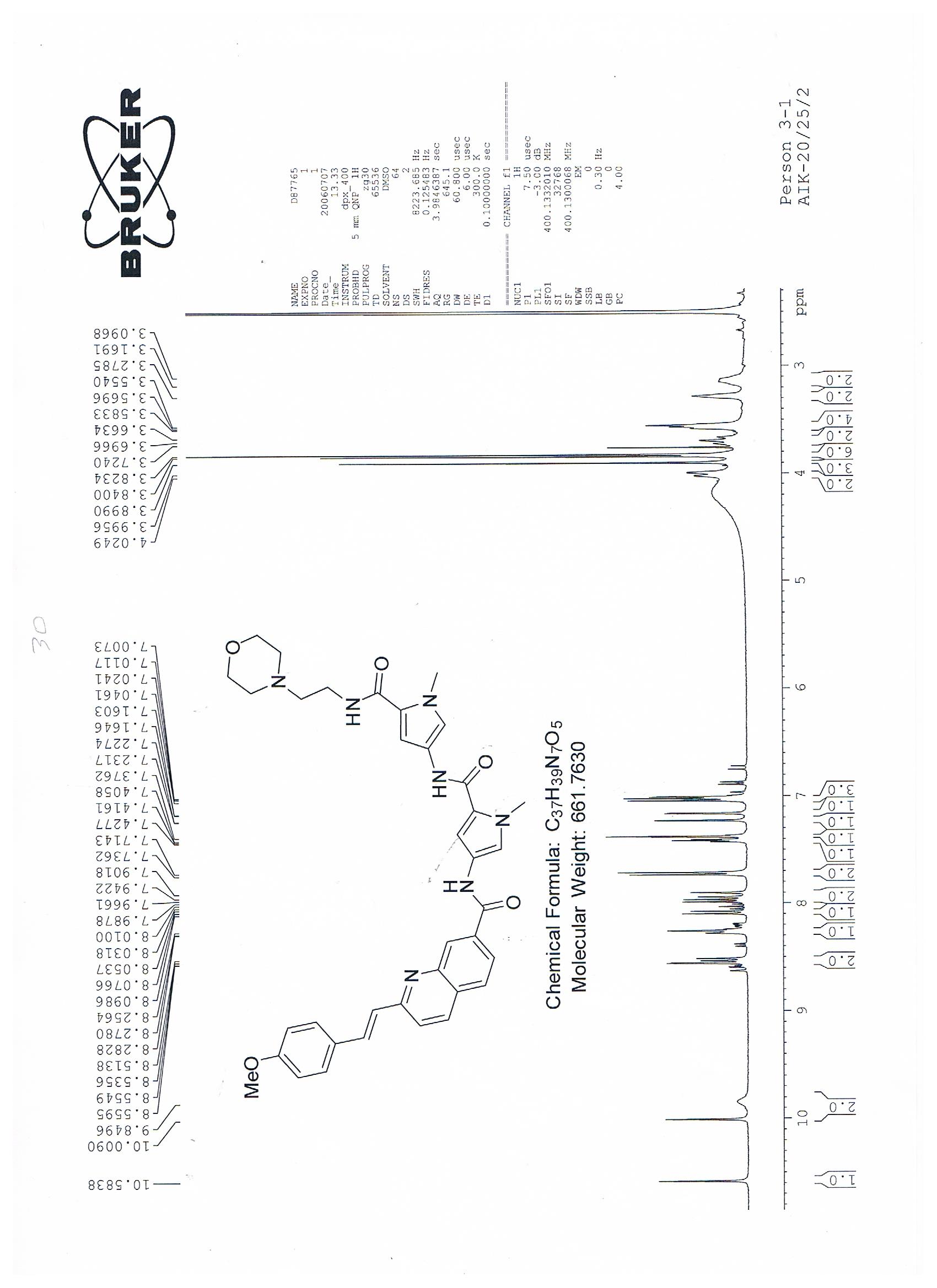


# 3. Novel MGB Fragments

Most of the MGB fragments used in the synthesis of the final MGBs in this paper have been published before. The following are the synthetic details for those fragments that are novel.

In the synthesis of compound **28**:

*Diethyl 2,1,3-benzothiadiazol-5-ylmethylphosphonate*

Triethylphosphite (363 mg, 2.18 mmol) was added to 5-(bromomethyl)-2,1,3-benzothiadiazole (250 mg, 1.09 mmol) and the reaction mixture was heated under reflux for 5h. Ethanol and excess triethylphosphite were removed *in vacuo* to give the required product as brown oil (312 mg, 100%).

IR (KBr): 2985, 1725, 1632, 1538, 1392, 1247, 1048, 1026, 826 cm^-1^.

^1^H NMR (DMSO-d_6_): 8.06(1H, d, J = 9.0 Hz), 7.98 (1H, d, J = 4.0 Hz), 4.03 (4H, dq, J = 7.1 Hz), 3.54 (2H, d, J = 22.1 Hz), 1.18 (6H, t, J = 7.1 Hz).

HRFABMS: Found: 286.0539 calculated for C_11_H_15_O_3_N_2_PS 286.0541

*Methyl 4-[(E)-2-(2,1,3-benzothiadiazol-5-yl)ethenyl]benzoate*

Diethyl 2,1,3-benzothiadiazol-5-ylmethylphosphonate (310 mg, 1.35 mmol) was dissolved in THF (5 mL, dry) then cooled to 0^o^C under nitrogen. Sodium hydride (100 mg, 60% suspension in oil, 2.50 mmol) was added portionwise with stirring. Methyl 4-formylbenzoate (192 mg, 1.17 mmol) was dissolved in THF (5 mL, dry) then added to the reaction mixture at 0^o^C under nitrogen with stirring. The reaction mixture was then left stirring at room temperature for 1h. Water was added at 0^o^C dropwise under nitrogen followed by dilute HCl until pH6. The reaction mixture was diluted with ethyl acetate and extracted. The water layer was extracted once more with ethyl acetate and the combined organic layers were dried (MgSO_4_) and the solvents were removed under reduced pressure. TLC showed one spot, ethyl acetate/n-hexane: 1/2) at R_F_=0.70. The crude product was dissolved in THF (5 mL) and applied to a column chromatography. Fractions containing the required product were collected and the solvents removed under reduced pressure to give the product as a pale yellow fine needles (260 mg, 75%), mp170-173^o^C.

IR (KBr): 2924, 1717, 1437, 1278, 1201, 1111, 946, 842, 803, 754 cm^-1^.

^1^H NMR (DMSO-d_6_): 8.16 (1H, s), 8.12 (2H, d, J = 9.2 Hz), 8.02 (2H, d, J = 8.4 Hz), 7.83 (2H, d, J = 8.4 Hz), 7.67 (2H, d, J = 3.2 Hz), 3.87 (3H, s).

HRFABMS: Found: 296.0620 calculated for C_16_H_12_O_2_N_2_S 296.0619

*4-[(E)-2-(2,1,3-benzothiadiazol-5-yl)ethenyl]benzoic acid*

Methyl 4-[(*E*)-2-(2,1,3-benzothiadiazol-5-yl)ethenyl]benzoate (240 mg, 0.89 mmol) was suspended in methanol (25 mL) to which sodium hydroxide solution [(500 mg, 12.5 mmol) in water (10 mL)] was added. The reaction mixture was heated under reflux for 3h. Some yellow solid material precipitated from the reaction mixture. This is probably the sodium salt of the carboxylic acid. Methanol was removed under reduced pressure then ether was added. After the extraction, the ether layer was removed and discarded. The water layer containing some suspension was cooled to 0^o^C and then hydrochloric acid (concentrated) was added dropwise with stirring until pH2. The yellow solid precipitate was filtered, washed with water and dried under reduced pressure to give the required product as yellow solid (115 mg, 46%), mp > 230^o^C.

IR (KBr): 3423, 2923, 1676, 1602, 1585, 1539, 1380, 1292, 1177, 946, 856, 806 776 cm^-1^.

^1^H NMR (DMSO-d_6_): 13.08 (1H, br), 8.19 (1H, d, J = 9.2 Hz), 8.13 (1H, d, J = 9.2 Hz), 8.03 (1H, s), 8.00 (2H, d, J = 8.4 Hz), 7.81 (2H, d, J = 8.4 Hz), 7.71 (2H, s).

HRFABMS: Found: 283.0543 calculated for C_15_H_11_O_2_N_2_S 283.0541

In the synthesis of compound **29**:

*Diethyl 2,1,3-benzoxadiazol-5-ylmethylphosphonate*

5-(Bromomethyl)-2,1,3-benzoxadiazole (250 mg, 1.17 mmol) and triethylphosphite (390 mg, 2.35 mmol) were heated under reflux for 5h. Excess triethylphosphite was removed *in vacuuo* to give the required product as brown oil (317 mg, 100%).

IR (KBr): 2985, 1728, 1632, 1538, 1443, 1392, 1247, 1026, 958, 826 cm^-1^.

^1^H NMR (DMSO-d_6_): 8.03 (1H, d, J = 9.3 Hz), 7.90 (1H, d, J = 4.5 Hz), 7.55 (1H, d, J = 9.3 Hz), 4.04 (4H, t, J = 7.0 Hz), 3.51 (2H, d, J = 22.5 Hz), 1.18 (6H, t, J = 7.0 Hz).

HRFABMS: Found: 271.0844 Calculated for C_11_H_16_N_2_O_4_P 271.0842.

*Methyl 4-[(E)-2-(2,1,3-benzoxadiazol-5-yl)ethenyl]benzoate*

Diethyl 2,1,3-benzoxadiazol-5-ylmethylphosphonate (310 mg, 1.15 mmol) was dissolved in THF (5 mL, dry) then cooled to 0^o^C under nitrogen. Sodium hydride (92 mg, 60% suspension in oil, 2.30 mmol) was added portionwise with stirring. Methyl 4-formylbenzoate (188 mg, 1.15 mmol) was dissolved in THF (5 mL, dry) then added to the reaction mixture at 0^o^C under nitrogen with stirring. The reaction mixture was then left stirring at room temperature for 1h. Water was added at 0^o^C dropwise under nitrogen followed by dilute HCl until pH6. The reaction mixture was diluted with ethyl acetate and extracted. The water layer was extracted once more with ethyl acetate and the combined organic layers were dried (MgSO_4_), filtered and the solvents were removed under reduced pressure. The crude product was dissolved in THF (5 mL) and applied to a silica gel column chromatography. The product was eluted with ethyl acetate/n-hexane (1/2), R_F_=0.50. Fractions containing the required product were collected and the solvents removed under reduced pressure to give the required product as pale yellow crystalline (300 mg, 93%), mp180-183^o^C.

IR (KBr): 2985, 1718, 1605, 1430, 1286, 1110, 1004, 949, 872, 802, 751 cm^-1^.

^1^H NMR (DMSO-d_6_): 8.12 (3H, m), 8.02 (2H, m), 7.83 (2H, m), 7.71-7.61 (2H, m), 3.87 (3H, s).

HRFABMS: Found: 281.0925 Calculated for C_16_H_13_N_2_O_3_ 281.0921

*4-[(E)-2-(2,1,3-Benzoxadiazol-5-yl)ethenyl]benzoic acid*

Methyl 4-[(*E*)-2-(2,1,3-benzoxadiazol-5-yl)ethenyl]benzoate (90 mg, 0.321 mmol) was dissolved in methanol (5 mL) to which sodium hydroxide solution (NaOH 200 mg, 5 mmol in water 10 mL) was added. The reaction mixture was heated under reflux for 3h. Methanol was removed under partial reduced pressure and the remaining solution was cooled to 0^o^C. Hydrochloric acid (conc.) was added dropwise with vigorous stirring until pH4. The yellow solid material was collected by filtration, washed with water and dried under reduced pressure at 50^o^C to give the required material (60 mg, 70%), mp > 280^o^C.

IR (KBr): 2912, 1678, 1608, 1426, 1316, 1286, 1179, 1007, 965, 952, 878, 847 cm^-1^.

^1^H NMR (DMSO-d_6_): 8.10 (3H, s & d, J = 4.2 Hz), 7.98 (2H, d, J = 8.3 Hz), 7.77 (2H, d, J = 8.3 Hz), 7.69 (1H, d, J = 16.5 Hz), 7.61(1H, d, J = 16.5 Hz).

HRFABMS: Found: 265.0616 C_15_H_9_N_2_O_3_ 265.0619

In the synthesis of compound **32**:

*4-{(E)-2-[4-(dimethylamino)phenyl]ethenyl}benzoic acid*

Prepared as in Van der Eycken et al (2002)

# 4. References

Erik Van der Eycken, Zhang Jidong, Amuri Kilonda, Frans Compernolle, Suzanne Toppet,  Georges Hoornaert, Mark Van der Auweraer, Carine Jackers, Wouter Verbouwe and Frans C. De Schryver, J. Chem. Soc., Perkin Trans. 2, **2002**, 928-937.
